# Cryptic glucocorticoid receptor-binding sites pervade genomic NF-κB response elements

**DOI:** 10.1038/s41467-018-03780-1

**Published:** 2018-04-06

**Authors:** William H. Hudson, Ian Mitchelle S. de Vera, Jerome C. Nwachukwu, Emily R. Weikum, Austin G. Herbst, Qin Yang, David L. Bain, Kendall W. Nettles, Douglas J. Kojetin, Eric A. Ortlund

**Affiliations:** 1 Department of Biochemistry, Emory University School of Medicine, Atlanta, Georgia 30322 USA; 2 Discovery and Developmental Therapeutics, Winship Cancer Institute, Atlanta, Georgia 30322 USA; 3 Department of Molecular Therapeutics, The Scripps Research Institute, Jupiter, Florida 33458 USA; 4 Department of Integrated Structural and Computational Biology, The Scripps Research Institute, Jupiter, Florida 33458 USA; 5 Department of Pharmaceutical Sciences, University of Colorado Anschutz Medical Campus, Aurora, Colorado, 80045​ USA; 6 Present Address: Department of Microbiology and Immunology, Emory University School of Medicine, Atlanta, Georgia 30322 USA; 7 Present Address: Department of Pharmacology and Physiology, Saint Louis University School of Medicine, Saint Louis, MO 63104 USA

## Abstract

Glucocorticoids (GCs) are potent repressors of NF-κB activity, making them a preferred choice for treatment of inflammation-driven conditions. Despite the widespread use of GCs in the clinic, current models are inadequate to explain the role of the glucocorticoid receptor (GR) within this critical signaling pathway. GR binding directly to NF-κB itself—tethering in a DNA binding-independent manner—represents the standing model of how GCs inhibit NF-κB-driven transcription. We demonstrate that direct binding of GR to genomic NF-κB response elements (κBREs) mediates GR-driven repression of inflammatory gene expression. We report five crystal structures and solution NMR data of GR DBD-κBRE complexes, which reveal that GR recognizes a cryptic response element between the binding footprints of NF-κB subunits within κBREs. These cryptic sequences exhibit high sequence and functional conservation, suggesting that GR binding to κBREs is an evolutionarily conserved mechanism of controlling the inflammatory response.

## Abstract (web summary)

Glucocorticoid receptor (GR) regulates immunity and inflammation but the mechanisms by which GR represses proinflammatory genes are still being debated. Here the authors use a multidisciplinary approach and show that GR binds to a cryptic site within genome-wide NFκB DNA response elements to repress pro-inflammatory genes.

## Introduction

Glucocorticoids (GCs) are a class of steroid hormones that are widely prescribed for inflammation-driven conditions such as asthma and arthritis^1^. GCs exert their effects by binding to the GC receptor (GR), a ubiquitously expressed nuclear receptor that drives both the activation and repression of its target genes^2^. Ligand-bound GR is able to antagonize the activity of immunogenic transcription factors such as nuclear factor-κB (NF-κB)^3^, AP-1^4,5^, and T-bet^6^, resulting in a potent attenuation of inflammation. Indeed, repression of pro-inflammatory genes such as interferon-γ by the GR is required to dampen immune responses that would be otherwise lethal^7,8^. Unfortunately, the therapeutic anti-inflammatory actions of GR are concomitant with a host of undesirable side effects that include skin atrophy, glaucoma, osteoporosis, adipogenesis, insulin resistance, and hypertension^9^. These opposing actions by GR have led to an intense—and largely unsuccessful—search for dissociated ligands that would separate its anti-inflammatory properties from its more malicious side effects at pharmacological doses^10^.

GR is normally sequestered in the cytoplasm and binding of GCs to its ligand-binding domain causes GR to translocate to the nucleus, where its DNA-binding domain (DBD) binds canonical activating GC response elements, or (+)GREs, which are pseudo-palindromic hexameric sequences containing two AGAACA (or similar) half-sites separated by 3 bp^11^. The agonist-bound conformation of the GR ligand-binding domain enables the recruitment of transcriptional coregulators^12^. An additional level of transcriptional regulation may occur through small variations in the canonical (+)GRE sequence, which slightly alters the conformation of DNA-bound GR^13,14^. In contrast, the repressive effects of GR on pro-inflammatory transcription factors are generally thought to be DNA independent^3,5,6,15^. In line with this hypothesis, GR has been shown in many circumstances to interact directly with NF-κB subunits and is thus believed to tether to NF-κB response elements (κBREs) without the use of its own DNA-binding capabilities^16^.

Recently, some GR-mediated transcriptional repression has been attributed to direct interactions of the receptor with DNA^17,18^. In 2011, the discovery of inverted-repeat negative GC response elements (nGREs) was found to mediate GC-induced repression of hundreds of genes^17^. Our subsequent crystallographic analyses demonstrated that the GR DBD binds these nGREs in a distinct orientation from (+)GREs^19^. Simultaneously, chromatin immunoprecipitation sequencing (ChIP-seq) studies demonstrated that GR occupies the genomic loci of some pro-inflammatory genes in the absence of pro-inflammatory signaling, indicating that the tethering model is insufficient to explain GR-mediated repression of NF-κB target genes^20^. Here we demonstrate that GR binds directly to κBREs and propose that direct GR–DNA interaction at these genomic loci are an important mechanism to repress the expression of pro-inflammatory genes.

## Results

### GR mutations preventing NF-κB repression dissociate (+)GRE and nGRE binding

The GR is one of five paralogous steroid receptors in humans, along with the estrogen, androgen, progesterone, and mineralocorticoid receptors. Recently, we demonstrated that GR is the only steroid receptor capable of binding to nGRE sequences and mediating transcriptional repression from these elements^21^. A single amino acid substitution unique to the GR evolutionary lineage, G425S, altered the receptor’s backbone conformation, enhancing its ability to bind DNA as a monomer^21^. The reverse mutation, S425G, renders the human GR incapable of binding nGREs^21^. Previously, the S425G mutation had been reported to ablate the ability of GR to repress NF-κB-driven transcription^22,23^. In line with these results, we found that the S425G mutation hindered the ability of full-length GR to repress a constitutively active reporter gene preceded by 400 bp of the *IL8* promoter, which contains a κBRE, but not a (+)GRE (Supplementary Figure 1a). We hypothesized this effect could be due to direct binding of GR to DNA, given the similar effect seen at DNA-dependent nGREs^17,19^.

We also examined the effects of two previously reported GR DBD mutations on binding to activating and repressive DNA elements. The GRdim mutant (A458T) disrupts the GR DBD’s dimer interface and is widely used in steroid receptor research^24^. The presumed inability of the GRdim mutant to bind DNA led to an initial observation that GR’s DNA-binding ability was not required for mouse survival or repression of NF-κB^23,25^. However, more recent reports suggest that both wild-type (WT) GR and the GRdim mutant are recruited in macrophages to κBREs, whereas only WT GR is recruited to conventional (+)GREs^26^. Similarly, the GTG3A GR mutant, which contains three mutations within the DBD, preferentially recognizes a thyroid hormone response element rather than (+)GREs, yet is capable of repressing NF-κB-driven transcription^27^. With in vitro-binding experiments, we find that the GTG3A mutation indeed has a lower affinity for (+)GRE sequences compared with WT GR, but its affinity for nGREs remains relatively unchanged (Supplementary Figure 1b-c). Together, these observations led us to hypothesize that direct GR–DNA interactions may be responsible for GC-mediated repression of pro-inflammatory genes.

### GR functional interaction with kBREs independent of TNF-α

To test the ability of GR to repress transcription of pro-inflammatory genes hosting κBREs within their promoter, we conducted RNA sequencing (RNA-seq) on A549 cells in the presence and absence of dexamethasone. Many NF-κB target genes were downregulated by dexamethasone treatment (Supplementary Figure 2a-b) and geneset enrichment analysis showed that dexamethasone-regulated genes had remarkable overlap with genes regulated by tumor necrosis factor (TNF)-α via NF-κB^28^ (*p* = 2 × 10^−45^). However, measuring transcriptional changes of NF-κB target genes in response to dexamethasone is not ideal due to the low abundance of many of these transcripts in the absence of pro-inflammatory signaling. Unfortunately, their induction by TNF-α would introduce the confounding factor of NF-κB activation, including the nuclear translocation of potential tethering factors and/or chromatin remodeling. To remove these confounding factors, we tested the ability of the GR double mutant K442A R447A to repress constitutively active reporters harboring ~ 150 bp of κBRE-containing promoters. The K442A R447A mutant lacks two key side chains critical for sequence-specific DNA recognition by GR at multiple response elements (refs. 13,19, and Supplementary Figure 2c). Although WT GR was able to repress several of these reporters, including *IL8*, *CCL2*, *RELB*, *PLAU*, and *ICAM1*, the K442A R447A mutant was generally unable to repress more than transfection with an empty vector (Supplementary Figure 2d-j). These results indicated that the DNA-binding ability of GR is critical for its ability to repress transcription of κBRE-containing promoters.

**Fig. 1:**
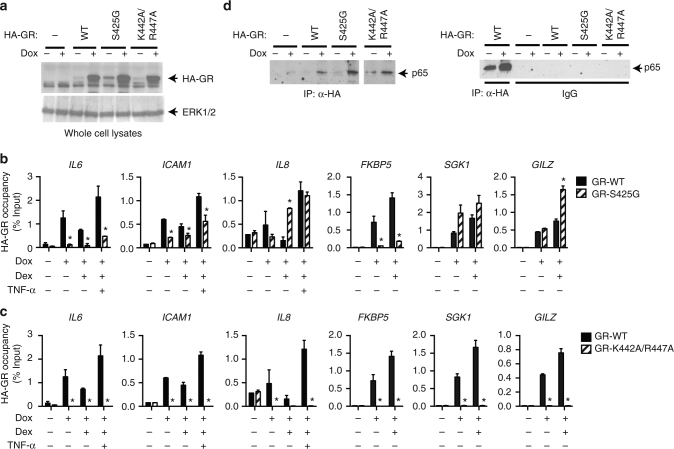
DNA-binding domain mutations alter recruitment of GR to native target genes. **a–c** HEK293T cells were transfected with the indicated expression plasmids. The next day, the cells were steroid-deprived and treated with or without 1 μg/ml doxycycline (Dox) for 24 h. **a** Whole cell lysates were analyzed by western blotting using anti-HA and anti-ERK1/2 antibodies. Uncropped blots are shown in Supplementary Figure 7. **b**, **c** HEK293T cells stimulated for 1 h with 100 nM dexamethasone (Dex) alone or in combination with 10 ng/ml TNF-α as indicated and analyzed by ChIP assay using anti-HA antibody. Shown as fold enrichment relative to vehicle (mean + SEM, *n* = 3) *Dunnett’s multiple comparisons test, *p*_adj_ < 0.05 relative to GR-WT transfectants. **d** HEK293T cells were transfected with p65/RelA, rtTA, and the inducible HA-GR- WT or -mutant expression plasmids. The next day, the cells were steroid-deprived and treated with or without 1 μg/ml Dox for 24 h and then stimulated with 10 ng/ml TNF-α for 1 h. Whole cell lysates were analyzed by IP using anti-HA antibody or normal (pre-immune) rabbit IgG, followed by western blotting using anti-p65 antibody

To understand how GR associates with native NF-κB target genes, we used a tetracycline-inducible system in HEK293T cells to express WT GR or DBD mutants, including the S425G and K442A R447A mutants. As these cells express GR endogenously, the exogenous receptors were detected using their N-terminal hemagglutinin (HA) epitope tag (Fig. 1a). Recruitment of exogenous GR to the native κBREs of NF-κB target genes (*IL6*, *IL8*, and *ICAM1*) and canonical (+)GREs of GC-induced genes (*FKBP5*, *SGK1*, and *TSC22D3/GILZ*; Supplementary Figure 3a) was then examined by ChIP assay using anti-HA antibody. Compared with WT GR, which was detected at every site examined, the S425G mutation reduced GR occupancy at specific NF-κB target genes (*IL6* and *ICAM1*, but not *IL8*) and one of the three GC-induced genes (*FKBP5* but not *GILZ* or *SGK1*), confirming that this mutation has gene-specific effects on GR’s DNA-binding ability and recruitment to target genes (Fig. 1b). Critically, the K442A R447A mutation was not recruited to canonical (+)GREs such as *FKBP5* or κBREs in the inflammatory genes (Fig. 1c), demonstrating that recognition of DNA—potentially in a sequence-specific manner—is required at both classes of sites.

As GR could be tethered to κBREs via protein–protein interaction with NF-κB, we tested whether these DBD mutations also interfere with this interaction. Similar to the WT receptor, GR K442A R447A and GR S425G associated with the p65/RelA subunit of NF-κB when co-expressed (Fig. 1d and Supplementary Figure 3b), indicating that protein–protein interaction between GR and NF-κB is not disrupted by the mutations and is not sufficient for GR-mediated transcriptional repression. Taken together, these results suggest that GR associates with native NF-κB target genes via direct DNA binding, a mechanism that is completely abolished by the GR K442 R447A mutation and modulated by the GR S425G substitution.

### GR binds directly to κBREs

**Fig. 2:**
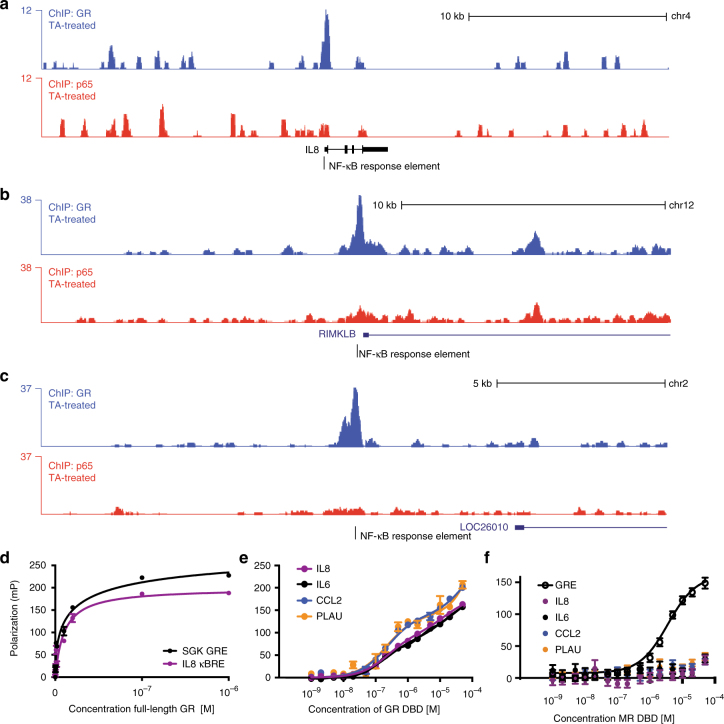
GR is recruited directly to NF-κB response elements. **a**–**c** ChIP-seq data from HeLa cells activated with the GR agonist triamcinolone acetonide shows activated GR is recruited to NF-κB response elements, even without the presence of p65 as a tethering factor. **d** In vitro fluorescence polarization binding experiments with full-length GR shows that its affinity for the IL8 NF-κB response element and SGK1 (+)GRE are similar (51 and 35 nM, respectively). **e** GR DBD binding to four human κBREs showed two-site binding curves, similar to that of GR DBD when binding to an nGRE^19^ (mean + SEM, *n* = 3; Supplementary Table 5). An extra sum-of-squares *F*-test was used to compare a two-site specific binding event to a one-site specific binding event; the resulting *p*-values are shown in the left column of the panel. **f** The mineralocorticoid receptor DBD binds to a (+)GRE, but not to any of the κBREs tested (mean + SEM, *n* = 3)

Multiple ChIP-seq studies have been performed to determine DNA sequence motifs bound by GR in cells. In many of these, κBREs are highly enriched at genomic GR-binding sites^20,26,29,30^. A recent study^30^ performed ChIP-seq on GR and the NF-κB subunit p65 in the presence of their respective ligands (or a combination), as well as with simultaneous  lentiviral short hairpin RNA knockdown of p65. Our reanalysis of that study’s data reveals that upon treatment with triamcinolone acetonide (TA), a synthetic GC, ~ 476 GR ChIP-seq peaks contain κBRE motifs, and the vast majority of these (83%) remain even upon p65 knockdown (Supplementary Figure 4). Genes nearby these peaks appear to have important roles in the inflammatory response, with moderate overrepresentation of terms such as regulation of apoptosis, response to lipopolysaccharide (LPS), and response to cytokine stimulus in Gene Ontology analysis (Supplementary Figure 4). Further, GR is recruited to many of these motifs, such as that in the *IL8* promoter, without corresponding occupancy by the NF-κB subunit p65 (Fig. 2a-c**)**. Given the receptor’s localization to κBREs in multiple ChIP-seq studies and the requirement of its DNA-binding residues shown here, we postulated that GR may be binding directly to κBREs, to repress pro-inflammatory transcription in a sequence-specific manner.

To test this hypothesis, we used fluorescence polarization to measure the ability of soluble, full-length GR to bind both the *IL8* κBRE and the *SGK1* (+)GRE (Fig. 2d). Remarkably, the affinity of full-length GR for the two elements was very similar, with *K*_d_s of 34 and 51 nM for the *SGK1* (+)GRE and the *IL8* κBRE, respectively. We were unable to purify enough full-length GR to test additional DNA elements so we tested the ability of the isolated GR DBD to bind the *IL8*, *CCL2*, and *PLAU* κBREs in vitro. The isolated GR DBD was able to bind to all three of these κBREs, with affinity similar to nGRE binding^19^ (Fig. 2e). The mineralocorticoid receptor DBD, which recognizes canonical GR binding sites^31^, is unable to bind any of the κBREs tested (Fig. 2f), suggesting that steroid receptor–κBRE interactions may be unique to GR, much similar to nGRE binding^21^.

**Fig. 3:**
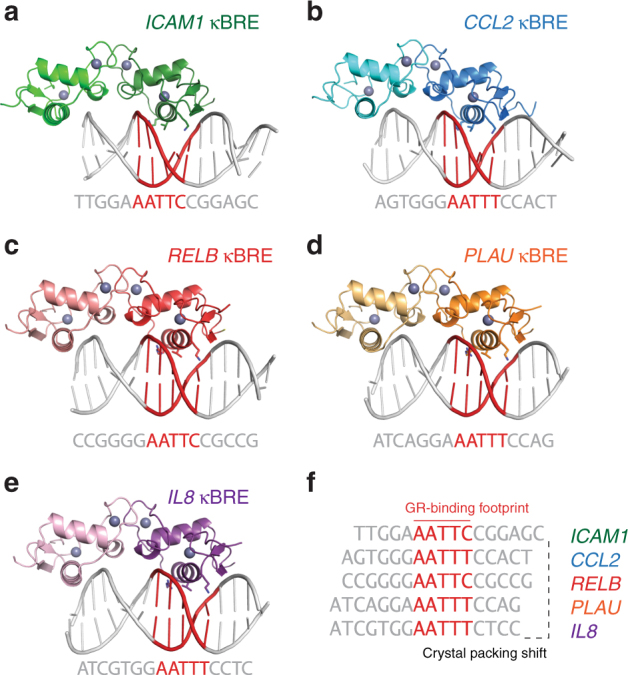
Five crystal structures of the GR DBD bound to NF-κB response elements reveal its binding footprint. **a**–**e** Overall representations of five GR DBD–NF-κB response element crystal structures. In each case, the GR DBD crystallized as a dimer in the asymmetric unit, with one monomer positioned over a DNA end stacking junction. The DNA sequence is shown below each structure, with the GR-binding footprint in red. **f** The DNA-binding footprints of GR are aligned, showing that crystal packing shifts in order to accommodate binding of GR to a specific AATTY sequence (Y, pyrimidine)

Given the direct interaction between the GR DBD and κBREs, we sought to uncover the structural mechanism by which GR bound these elements. Using X-ray crystallography, we solved five crystal structures of the GR DBD bound to κBREs from the *CCL2*, *ICAM1*, *IL8*, *PLAU*, and *RELB* promoters, at resolutions ranging from 1.85 to 2.30 Å (Table 1, Fig. 3a-e and Supplementary Figure 5). In all of these crystal structures, the GR DBD formed a dimer within the asymmetric unit (Fig. 3a-e). However, in all structures, one DBD monomer was consistently located above the end-stacking junction of the pseudo-continuous DNA helix formed by crystal packing. Therefore, it is likely that sequence-specific contacts to κBREs are made with only one GR monomer.

**Table 1:** Data collection and refinement statistics

	GR DBD–*IL8* κBRE	GR DBD–*ICAM1* κBRE	GR DBD–*PLAU* κBRE	GR DBD–*RELB* κBRE	GR DBD–*CCL2* κBRE
Data collection					
Space group	P2_1_2_1_2_1_	P2_1_2_1_2_1_	P2_1_2_1_2_1_	P2_1_2_1_2_1_	P2_1_2_1_2_1_
Cell dimensions					
*a*, *b*, *c* (Å)	39.2, 97.2, 103.3	39.1,97.1, 104.0	39.9, 95.4, 103.5	39.2, 97.0, 103.3	39.1, 97.4, 103.9
Resolution (Å)	34.27–1.85 (1.92–1.85)	50.00–2.17 (2.25–2.17)	50.00–2.15 (2.23–2.15)	50.00–2.25 (2.33–2.25)	48.70–2.20 (2.28–2.20)
*R*_merge_	10.9	6.9	19.1	9.8	9.5
*I*/*σI*	14.0 (2.8)	20.0 (1.8)	16.2 (1.7)	19.8 (1.9)	20.9 (2.1)
Completeness (%)	88.4 (74.7)	96.6 (91.6)	100.0 (100.0)	98.1 (84.9)	98.3 (87.8)
Redundancy	4.4 (2.3)	5.1 (4.3)	7.1 (6.7)	6.3 (2.9)	6.7 (5.1)
Refinement					
Resolution (Å)	1.85	2.40	2.20	2.25	2.20
No. reflections	30412	15785	20243	19106	20451
*R*_work_/*R*_free_	22.0/24.2	25.7/28.4	23.6/26.6	20.4/22.8	23.3/27.1
No. atoms					
Protein/DNA	1774	1767	1774	1777	1808
Water	96	19	51	75	29
B-factors					
Protein/DNA	51.5	97.2	50.7	51.3	58.0
Water	50.0	61.1	48.6	46.2	46.2
R.m.s. deviations					
Bond lengths (Å)	0.005	0.008	0.005	0.004	0.011
Bond angles (°)	0.5	1.17	0.85	0.72	1.18
Ramachandran					
Favored (%)	98	92	96	96	94
Outliers (%)	0	1	0	0	0
PDB ID	5E69	5E6D	5E6A	5E6B	5E6C

*Data collected from a single crystal; values in parentheses are for highest-resolution shell.

### GR recognizes κBREs in a sequence-specific manner

An interesting feature of the five crystal structures in complex with κBREs is the common binding footprint for the central GR DBD monomer (Fig. 3f). In each structure, GR DBD recognizes an AATTY sequence, with Y representing a pyrimidine base. Although each of the five structures were solved with a 16 bp oligonucleotide, the DNA packs in three distinct conformations driven by GR specificity for the AATTY sequence (Fig. 3f). PISA analysis^32^ indicates that the free-energy gain on formation of the AATTY-bound GR DBD monomer and the *IL8* κBRE is a very favorable − 8.7 kcal mol^−1^, similar to the change seen upon GR-nGRE binding.

**Fig. 4:**
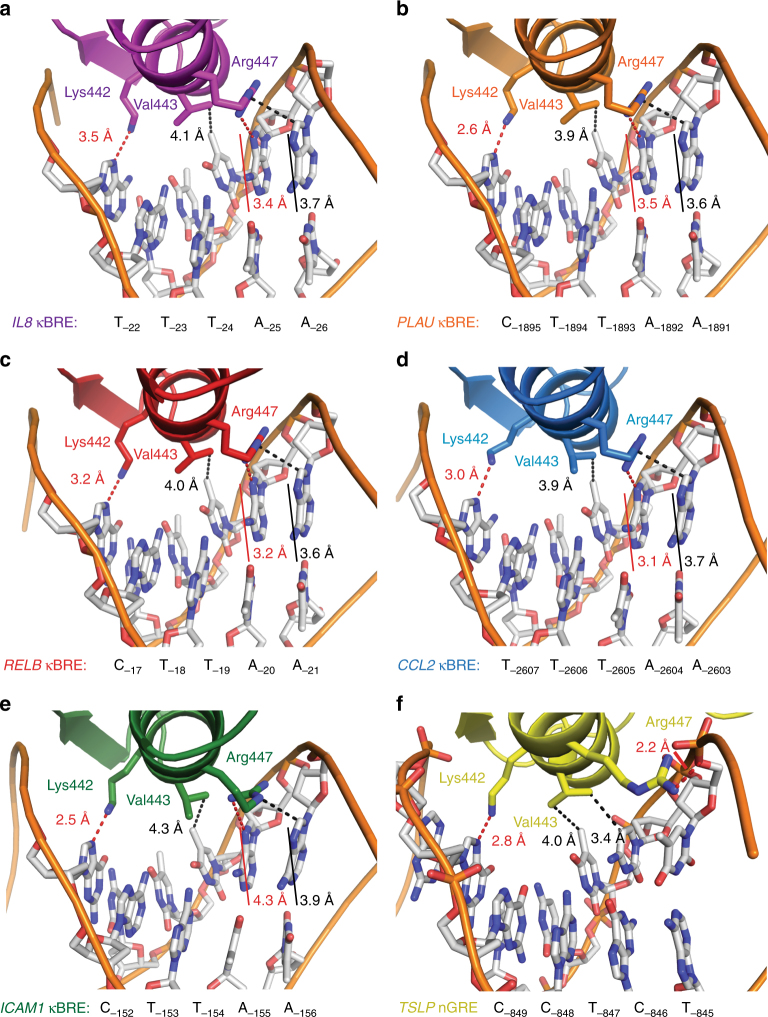
The GR DBD makes sequence-specific contacts with NF-κB response elements. **a**–**e** Sequence-specific contacts between the GR DBD and the *CCL2*, *IL8*, *PLAU*, *RELB*, and *ICAM1* NF-κB response elements. For comparison, sequence-specific contacts between the GR DBD and the *TSLP* nGRE are shown in **f**

Examination of the GR DBD–DNA interface reveals that GR recognizes the AATTY sequence in a specific manner (Fig. 4). Through the side chains of Lys442, Val443, and Arg447, the GR DBD makes contacts with four of the five bases within the AATTY motif. Arg447 recognizes the first two bases of this sequence: its guanidino group makes van der Waals contacts with the first adenine and a terminal amine forms a hydrogen bond with the second adenine in the motif (Fig. 4a-e). Val443 participates in a van der Waals interaction with the C7 of the thymine in the central A:T base pair; this distance is constant across the five crystal structures (3.8–4.3 Å; Fig. 4a-e). Finally, Lys442 makes a moderately strong, electrostatic hydrogen bond with the purine residue opposite the final base in the AATTY motif (Fig. 4a-e). These interactions are similar to GR’s contacts at the *TSLP* nGRE, with the exception of the position of Arg447 (Fig. 4f).

**Fig. 5:**
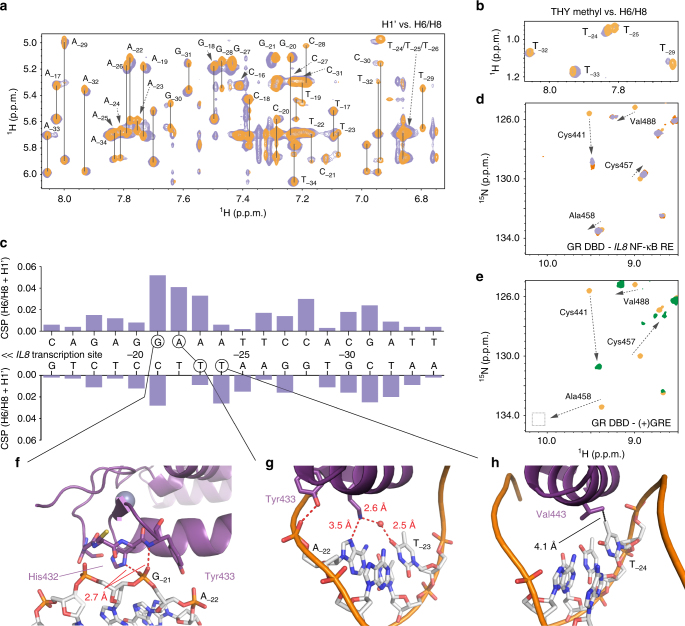
NMR reveals an interaction footprint of the GR DBD-*IL8* NF-κB response element complex that is consistent with crystal structures. **a**, **b** 2D homonuclear [^1^H,^1^H]-NOESY NMR data for *IL8* NF-κB response element alone (orange) or bound to GR DBD (2.3:1 molar ratio, purple). **c** NMR chemical shift perturbation analysis for data shown in **a**. **f–h** Show insets of highly perturbed bases. **d** 2D [^1^H,^15^N]-HSQC NMR analysis of ^15^N-labeled GR DBD (orange) and the same bound to *IL8* NF-κB response element (0.44:1 molar ratio, dark orange; 2.3:1 molar ratio, purple). **e** 2D [^1^H,^15^N]-HSQC NMR analysis of ^15^N-GR DBD (orange) and the same bound to (+)GRE consensus DNA response element (green). **f** Base G_-21_, which makes two close contacts with the GR DBD, is highly perturbed upon formation of the complex. The same is true of bases A_-22_ (**g**) and T_-24_ (**h**)

To test whether recognition of the AATTY motif is required for GR-κBRE binding in solution, we performed nuclear magnetic resonance (NMR) footprinting analysis to map the interaction between GR DBD and the *IL8* κBRE (Fig. 5a-e). Two-dimensional (2D) homonuclear [^1^H,^1^H]- nuclear Overhauser effect spectroscopy (NOESY) NMR analysis reveals that the in-solution GR DBD-binding footprint on the *IL8* κBRE is consistent with the crystal structure (Fig. 5a-c). Furthermore, the NMR data suggest the nucleotides near the AATTY sequence are most perturbed (Fig. 5c). The largest chemical shift perturbation occurred at guanine-21, which is directly adjacent to the first adenine of the AATTY motif. This is in strong agreement with the crystal structure of the GR DBD–*IL8* κBRE structure, as the GR DBD makes two close interactions (2.7 Å) with the DNA backbone at this position (Fig. 5f). The next two largest chemical shift perturbations upon GR DBD binding occurred within the AATTY motif, including adenine-22, which is directly contacted by Lys442 of the GR DBD (Fig. 5g). Crucially, thymine-24, which is contacted directly by Val443 of the GR DBD (Fig. 5h), also had a significant chemical shift perturbation upon GR binding (Fig. 5b, c).

On the protein, 2D [^1^H,^15^N]-heteronuclear single quantum coherence (HSQC) NMR analysis reveals that binding of *IL8* κBRE to ^15^N-labeled GR DBD causes large chemical shift perturbations for residues that contact DNA, such as Cys441 and Val488 (Fig. 5d). When GR DBD binds to (+)GRE DNA as a homodimer, this causes large chemical shift perturbations in the dimerization loop (or “D-loop”) residues, such as Ala458 and Gly459^14^ (Fig. 5e). However, when bound to the *IL8* κBRE, these “D-loop” residues were not affected (Fig. 5d), confirming that when GR DBD binds to this DNA sequence it does bind as a “D-loop” engaged dimer. This is consistent with recent reports that a monomeric full-length GR protein carrying mutations at both the DBD and LBD dimerization interfaces remains capable of repressing NF-κB activity^33^, as well as our previous observations by NMR that monomeric nGRE interactions do not perturb the “D-loop”^21^.

### Cryptic GR-binding sites within κBREs are highly conserved

**Fig. 6:**
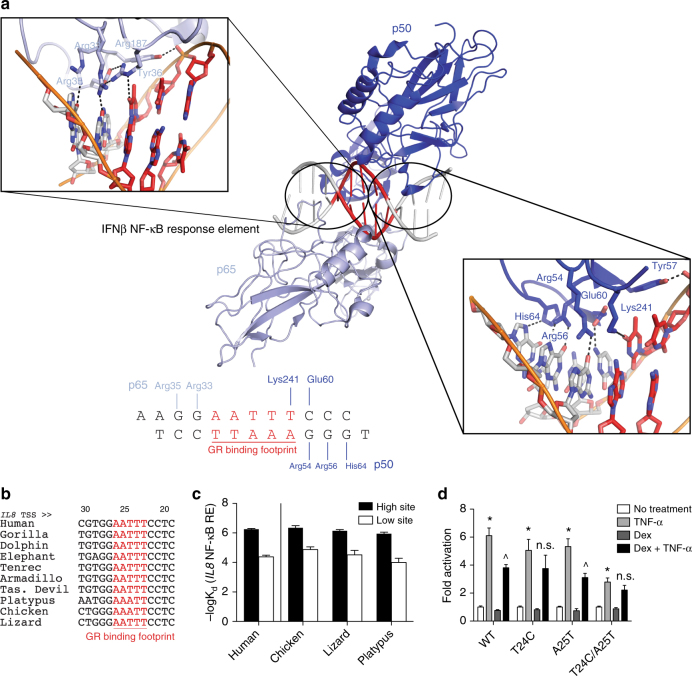
The GR DBD binds to a cryptic sequence in the spacer of NF-κB response elements. **a** Crystal structure of the p50/p65 NF-κB heterodimer bound to its cognate response element in the IFNβ promoter^71^. The GR-binding footprint (AATTY sequence) at this response element is shown in red. At this element (and many of its other response elements), NF-κB largely makes sequence-specific contacts with the regions flanking the AATTY motif (see insets). However, despite the lack of sequence-specific contacts by NF-κB in this spacer region, the AATTY motif is highly conserved; the conservation of the AATTY motif within the *IL8* NF-κB response element is shown in **b**. **c** In addition, the GR DBD can bind to the *IL8* NF-κB response element from divergent species with nearly-identical affinities. **d** Finally, mutation of central, conserved bases within the AATTY motif of the *IL8* promoter affects the ability of dexamethasone to repress transcription from this response element in HeLa cells. *p < 0.001 of TNF-α vs. no treatment; ^*p* < 0.001 of TNF-α vs. TNF-α + Dex; NS, no significance between TNF-α vs. TNF-α + Dex. Statistics are two-way ANOVA with Tukey’s post-hoc test.

NF-κB binds as a homo- or heterodimer of Rel homology domain-containing proteins to κBREs by specifically recognizing two binding footprints surrounding a central spacer in which the AATTY site is found. In a p50/p65 heterodimer, the central base pairs of the κBRE are not specifically bound by NF-κB itself (Fig. 6a)^34^. Despite this lack of sequence discrimination by NF-κB, an AATTY motif is overrepresented in the spacer region in its genomic response elements^35^. No satisfactory explanation for this over-representation has been provided by structural analyses of NF-κB binding alone^35^, and a recent SELEX study to determine the optimal NF-κB-binding motif revealed little sequence preference by the protein at this spacer sequence^36^. Given our findings that GR recognizes a cryptic AATTY motif present within κBREs, we propose that this motif is widely prevalent to ensure that these elements can be bound and repressed by GR. Supporting this hypothesis, the AATTY motif is present in many NF-κB-responsive genes that are regulated by dexamethasone (Supplementary Table 1**)**.

Furthermore, as GR, but not NF-κB, recognizes the AATTY motif within the central spacer sequence, we expected this motif to be highly conserved despite a lack of evolutionary pressure from NF-κB DNA-binding requirements. Indeed, we found that the AATTY motif is extremely well conserved—often more so than the bases that contact NF-κB (Supplementary Table 2). In a remarkable example, the AATTT motif or its reverse complement at the *IL8* κBRE is perfectly conserved from mammals to reptiles (Fig. 6b). This element also exhibits strong functional conservation, as human GR DBD retains the ability to bind the *IL8* κBRE from multiple species (Fig. 6c). Finally, mutation of one or more of these conserved bases from the human *IL8* κBRE reduced or ablated the ability of dexamethasone to repress the human *IL8* promoter (Fig. 6d), supporting both a sequence-specific recognition of the element by GR as well as a functional role for these highly conserved spacer bases at the *IL8* κBRE. Taken together, our results demonstrate that GR binds a conserved, cryptic sequence within a subset of κBREs to repress pro-inflammatory transcription.

### GR requires multiple coregulators to suppress cytokine genes

**Fig. 7:**
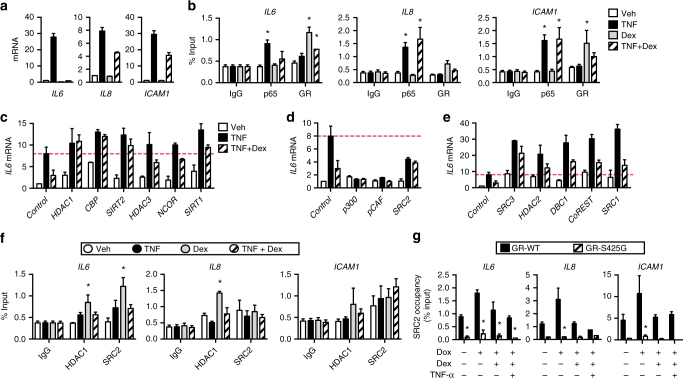
Coregulators are essential for GR-mediated repression of NF-κB. **a**, **b** Effects of dexamethasone (Dex) on NF-κB target genes. Steroid-deprived MCF-7 cells were stimulated with vehicle, 10 ng/ml TNF-α, 100 nM dexamethasone, or both. **a** After 4 h, total RNA was isolated and analyzed by qPCR. **b** The cells were fixed after 1 h and promoter occupancies at *IL6*, *IL8*, and *ICAM1* were compared by ChIP assay using anti-p65 NF-κB or anti-GR antibody (mean ± SEM; *n* = 3). *Dunnett’s multiple comparisons test, *p*_adj_ < 0.05 relative to vehicle-treated cells. **c**–**e** MCF-7 cells transfected with control or the indicated target siRNAs were stimulated with 10 ng/ml TNF-α alone or in combination with 10 nM dexamethasone for 2 h. *IL6* mRNA levels (mean ± SEM; *n* = 3) determined by qPCR are shown relative to levels in control siRNA transfectants stimulated with the vehicle. **d** Genes required for TNF-α-induced activity. **e** Genes that globally suppress TNF-α-induced activity. **f** GR coregulators are recruited to native nGREs/κBREs. Steroid-deprived MCF-7 cells were stimulated with vehicle, 10 ng/ml TNF, 100 nM dexamethasone, or a combination of TNF and dexamethasone, and fixed after 1 h. Promoter occupancies at *IL6*, *IL8*, and *ICAM1* were compared by ChIP assay using anti-HDAC1 or anti-SRC2 antibody (mean ± SEM; *n* = 3). *Dunnett’s multiple comparisons test, *p*_adj_ < 0.05 relative to vehicle-treated cells. **g** GR modulates coregulator recruitment at native nGREs/κBREs. HEK293T cells were transfected with the indicated expression plasmids. The next day, the cells were steroid-deprived and treated with or without 1 μg/ml doxycycline (Dox) for 24 h. The cells were then stimulated for 1 h with 100 nM dexamethasone alone or in combination with 10 ng/ml TNF and analyzed by ChIP assay using **e** anti-HDAC1 or **f** anti-SRC2 antibody. Shown as % input (mean + SEM, *n* = 3). *Sidak’s multiple comparisons test, *p*_adj_ < 0.05 relative to GR-WT transfectants

In MCF-7 cells, dexamethasone repressed TNF-α-induced expression of pro-inflammatory genes to different extents (Fig. 7a). To determine whether these differences reflect the ability of GR to compete against NF-κB for distinct nGREs/κBREs, we examined the recruitment of endogenous GR and the NF-κB subunit, p65, to the *IL6*, *IL8*, and *ICAM1* promoters in MCF-7 cells stimulated for 1 h with 10 ng/ml TNF-α, 100 nM dexamethasone, or both. Compared with vehicle-stimulated cells, TNF-α and dexamethasone increased recruitment of p65 and GR, respectively (Fig. 7b). In dexamethasone-treated cells, TNF-α stimulation reduced GR recruitment to some of these sites, demonstrating that NF-κB signaling was not required for GR binding. Dexamethasone also blocked NF-κB recruitment in a gene-selective manner. In TNF-stimulated cells, dexamethasone abolished recruitment of p65 to *IL6*, without affecting p65 recruitment at *IL8* or *ICAM1* (Fig. 7b). These results indicate that other factors, in addition to sequence-specific κBRE binding by GR, are required to explain dexamethasone-dependent repression of NF-κB target genes.

To gain further insight into the molecular requirements of GR-mediated suppression of NF-κB activity, we used a small-scale small interfering RNA (siRNA) screen targeting 22 coregulator genes, to determine which are required for suppression of the *IL6* gene in MCF-7 cells. These siRNAs have been previously shown to knockdown expression of their targets^37^, which include nuclear receptor corepressors, histone deacetylases, and the nuclear receptor coactivators, SRC1–3 (also known as NCOA1, NCOA2, and NCOA3), which have intrinsic lysine acetyltransferase (KAT) activity, and are primary scaffolds for recruitment of other KATs such as CBP, p300, and PCAF, and assorted coregulators.

We identified a number of coregulators whose knockdown partially or fully prevented dexamethasone from suppressing TNF-α induction (Fig. 7c). These include corepressors, NCoR, HDAC1, and HDAC3, but also the coactivator CBP. SIRT1 and SIRT2, NAD^+^-dependent coregulators that deacetylate p65 Lys320^38,39^, were both required for dexamethasone-mediated suppression of *IL6* (Fig. 7c). We also identified three coregulators required for TNF-α-induced activation of *IL6*, including p300 and pCAF (Fig. 7d). SRC2 has been previously reported as required for GR-mediated anti-inflammatory effects^40^, but we observed that it had a dual role. SRC2 knockdown both reduced TNF-α-dependent activation and abolished dexamethasone-dependent suppression of *IL6*, demonstrating that it has roles in both activation and suppression at this locus.

There were also a set of coregulators whose knockdown led to global increases in *IL6* activity with both vehicle and TNF-α treatment. Deleted in breast cancer-1 (DBC1/CCAR2) associates with SIRT1 and inhibit its deacetylase activity^41,42^. DBC1 was not required for GR-mediated suppression of *IL6*, but knockdown of DBC1 led to a TNF-α- and dexamethasone-independent increase in *IL6* expression, similar to effects of knockdown of HDAC2 and CoREST corepressors (Fig. 7e). Knockdown of SRC1 and SRC3 also globally increased IL6, but SRC3 displayed an additional role in dexamethasone-mediated suppression. Thus SRC1-3 family members displayed some distinct and some overlapping roles in integrating inflammatory and GC responses.

In contrast, silencing mediator of retinoid and thyroid hormone receptor (SMRT) or ligand dependent nuclear receptor corepressor (LCoR) siRNA did not increase *IL6* expression or relieve the inhibitory effect of dexamethasone (Supplementary Figure 6), suggesting that SMRT and LCoR did not contribute to repression of *IL6*. Further, siRNAs against the lysine demethylase LSD1/KDM1A, the lysine methyltransferase GLP/KMT1D, and the corepressor CTBP1, which are found in complex with HDAC1 and CoREST^43,44^, also did not have an effect on *IL6* expression (Supplementary Figure 6). Taken together, these results suggest that GR requires a specific subset of coregulators, including SRC2, SRC3, CBP, HDAC1, and SIRT2 to mediate ligand-dependent suppression of NF-κB-mediated transcription at the *IL6* gene.

### GR modulates the recruitment of coregulators at κBREs

We examined recruitment of HDAC1 and SRC2 to the promoter regions of the *IL6*, *IL8*, and *ICAM1* genes in MCF-7 cells by ChIP assay. All of the treatments induced recruitment of HDAC1 and SRC2 to the *IL6* promoter, but dexamethasone induced the greatest recruitment (Fig. 7f), consistent with direct binding by GR in the absence of NF-κB signaling. Dexamethasone also induced recruitment of HDAC1 to *IL8*, but did not induce recruitment of SRC2 to *IL8* or *ICAM1* (Fig. 7f), demonstrating that GR controls coregulator recruitment at native κBREs in a gene-specific manner. To test the role of monomeric GR binding, we compared the recruitment of endogenous SRC2 in HEK293T cells transfected with tetracycline-inducible expression plasmids for HA-tagged GR-WT or the mutant with reduced affinity for nGREs, GR-S425G. SRC2 was recruited to the *IL6*, *IL8*, and *ICAM1* promoters, and this recruitment was completely abolished by the GR-S425G mutation that displays reduced affinity for nGREs (Fig. 7g). Taken together, these results are consistent with a model where GR controls recruitment of coregulators required for GC-dependent suppression of NF-κB target genes by binding directly to nGREs/κBREs via DBD-mediated, sequence-specific recognition of DNA.

## Discussion

A defining feature of the inflammatory response is the integration of signals from a variety of pathways at the promoters of cytokine and chemokine genes, including direct binding of NF-κB, AP-1, IRF, STAT, and Creb family members. Opposing signaling through these pathways by GCs is strictly required to avoid lethal immune overactivation in response to infection or other inflammatory stimuli^7,8^. Despite the importance of GR signaling, the mechanism of inflammatory gene repression by GCs has remained controversial. Here we show that GR binds directly to a highly conserved, cryptic sequence within κBREs at these promoters, such as those of *IL8*, *CCL2*, and *ICAM1*.

Through reanalysis of ChIP-seq data, we find that the κBRE motif exists at 5%–10% of GR-bound sites after GC treatment alone, in line with other studies^20,29,30^. As GR activation does not alter the subcellular localization of NF-κB^45-47^, this data suggested the possibility of direct interaction between GR and NF-κB-driven promoters. Following NF-κB activation, the κBRE is present at ~ 25% of GR-occupied sites^30^. In contrast to the tethering hypothesis, we show here that GR’s DNA-binding ability is required for recruitment to these genes. This is in line with a previous study that demonstrated that some GR-binding sites, which did not co-occupy with NF-κB or AP-1, are only accessible and bound upon LPS treatment^20^.

The model we propose—that GR binds κBREs directly—represents a shift in the current models of GR action. Thus, it is worthwhile to re-examine previous studies in light of the hypothesis we propose here. Hundreds of studies have been performed with GR mutant proteins and their conclusions are quite complex^24^. At least four studies have demonstrated that GR activation affects the DNA binding of NF-κB^3,46,-48^, although this was not observed in at least three other reports^45,49,50^, whereas our data demonstrates that these effects are gene selective. Our model predicts that GR and NF-κB compete directly for the same binding site at some loci. However, experiments using GR variants demonstrate that two proteins binding the same response element can undergo dynamic exchange with short protein–DNA residency times and, in fact, do not compete^51^. Many studies, including ours, have shown that GR and the p65 subunit of NF-κB interact^3,27,47,50,52^. However, the existence of GR mutants, such as the K442A R447A mutant described here, able to bind NF-κB yet deficient in its repression demonstrate that a GR-NF-κB interaction is not sufficient for GC-mediated repression of NF-κB^50,52^. In line with our results, the DNA-binding domain of GR has been previously shown to be crucial for repression of NF-κB^27,47,53^.

Although we propose that direct GR binding to κBREs is an important mechanism of GC action, it is likely to be that multiple mechanisms govern GR-mediated suppression of inflammation and possible that multiple mechanisms act on any given gene. Some GR ligands affect multiple functions of the protein. For example, selective GR modulators have been reported to differentially affect repression of AP-1 and NF-κB^54^. Newly discovered DNA-binding motifs mediate some of the transcriptional repression by GR, as nGREs may represent a genome-wide class of GR binding sites^17^. Importantly, ChIP-seq studies have validated the widespread role of monomeric GR in GC function. Finally, ChIP-seq studies have also found similar GR-bound DNA sequences near genes both repressed and activated by GC treatment^20^. Although this finding indicates that DNA sequence and/or oligomerization state^33^ may not be sufficient to predict GR action at a particular site, it also confirms that the current tethering model of protein–protein interactions between GR and NF-κB is insufficient to explain all GC-mediated transcriptional responses. Here we propose that direct, sequence-specific interactions between GR and some κBREs are critical for transcriptional repression previously explained exclusively by tethering.

Current models of transcriptional regulation suggest a signaling pathway controlled by ordered, cyclical patterns of protein recruitment. However, this occurs in a highly dynamic, stochastic manner, with rapid and transient assembly of different complexes, a subset of which facilitates active transcription^55^. This suggests a model whereby GR samples the *IL6* promoter in several structurally distinct modes that involve both direct binding to the κBRE and tethered binding via protein–protein interaction with DNA-bound transcription factors such as p65, and various coregulator complexes to disrupt NF-κB-mediated transcription.

## Methods

### Analysis of ChIP-seq data

Previously reported ChIP-seq reads^30^ were downloaded from the NCBI Sequence Read Archive (SRA): samples GSM604648, GSM604649, GSM604651, GSM604656, GSM604657, GSM604662, and GSM604663. These were aligned to the human GRCh38 genome using Bowtie2^56^. ChIP-seq peaks were called using MACS 1.4^57^ with *P*-value cutoff of 1 × 10^−5^. For both p65 and GR, dimethyl sulfoxide-treated samples (GSM604648, GSM604656, and GSM604662) were used as negative controls. bedtools intersect^58^ was used to determine shared peaks among samples with a minimum overlap of 1 bp. UCSC Table Browser was used to obtain genomic sequences, and were considered to contain an κBRE if their genomic sequences contained at least one site matching the NF-κB (Hsapiens-JASPAR_2014-NFKB1-MA0105.3) JASPAR motif with a score of 90% or greater, as determined using the Biostrings package in Bioconductor^59^. For Gene Ontology analyses, the nearest transcription start site to each peak was determined with the ChIPpeakAnno package^59^ and results input to the DAVID ontology server^60^.

### RNA-sequencing

Approximately 10^6^ A549 cells cultured in complete Dulbecco’s modified Eagle’s medium (DMEM) were transfected with 2 μg pcDNA3.1 with FuGene HD, according to manufacturer’s protocols. Twenty-four hours after transfection, cells were treated with 100 nM dexamethasone or vehicle control (ethanol). Twenty-four hours after treatment, RNA was isolated with the Qiagen RNEasy kit, according to manufacturer’s protocols. Library preparation and sequencing were performed by the Baylor University Genomic and RNA Profiling Core. Reads were mapped to the human genome with tophat2^61^ to the *Homo sapiens* reference genome (GRCh38). Cuffdiff was used to compare gene expression between the dexamethasone-treated and vehicle-treated groups, with a FDR of 0.01^62^. Sequence reads for this experiment have been deposited in the SRA under BioProject accession PRJNA314815.

### Protein expression and purification

DBDs were expressed and purified as previously described^63^: residues 417–506 of the human GR (GenBank ADP91252) and residues 593–671 of the human MR (GenBank AAA59571.1) were expressed as an N-terminal 6 × -His fusion followed by a TEV protease cleavage site. *Escherichia coli* BL21(DE3)pLysS cells were induced with 0.3 mM isopropyl β-d-1-thiogalactopyranoside for 4 h at 30 °C after reaching an OD_600_ of ~ 0.6. Proteins were purified via affinity chromatography (HisTrap) followed by gel filtration in 100 mM NaCl, 20 mM Tris-HCl pH 7.4, and 5% glycerol. Protein was concentrated to 4 mg/ml, flash frozen in liquid N_2_, and stored at − 80 °C until use. ^15^N-GR DBD was expressed in *E. coli* BL21(DE3) pLysS cells with ^15^NH_4_Cl as the sole nitrogen source, purified as described above. The 6 × -His tag was cleaved with TEV protease overnight at 4 °C, passed through an NiNTA column, and the flow through containing purified ^15^N-GR DBD was collected and verified to be > 99% pure by SDS-polyacrylamide gel electrophoresis (PAGE). Residues 1–777 of human GR were expressed as an N-terminal 6 × -His fusion in baculovirus-infected Sf9 cells^64^. Cells were treated with 1 μM TA for 24 h post infection and then collected 24 h later. FL GR from the nuclear-localized fraction was purified via affinity chromatography (Ni-NTA agarose resin), followed by gel filtration in 500 mM NaCl, 20 mM Tris-HCL pH 8.0, 10 mM b-ME, 10 mM TA, and 10 % glycerol. The protein was then concentrated to ~ 1 mg/ml by ion-exchange chromatography, and flash frozen and stored in liquid N_2_. FL GR was judged to be > 95% pure by SDS-PAGE analysis.

### Reporter gene assays

For Supplementary Figure 2, Reporter gene assays were performed as previously reported^19^: indicated amounts of receptor plasmid (in the pcDNA3.1 vector), 50 ng of a pGL3 plasmid (Firefly luciferase) containing the indicated promoter (see below) constitutively driven by the SV40 promoter and enhancer, and 10 ng of a constitutively active *Renilla* luciferase under the control of the pRL-TK promoter were transfected into HeLa cells (gift from Haian Fu, Emory University) with FuGene HD (Promega) according to the manufacturer’s protocol. Amounts of reporter vector were constant in all titrations of receptor plasmid. Twenty-four hours after transfection, cells were treated with 1 μM dexamethasone, and 24 h following treatment firefly and *Renilla* luciferase activities were read using a Biotek Synergy plate reader and the Dual-Glo Luciferase Assay System (Promega), according to the manufacturer’s protocol. In Fig. 5d, 50 ng of the *IL8* promoter (or the indicated mutant) in the pGL3 basic vector and 10 ng of a constitutively active *Renilla* luciferase under the control of the pRL-TK promoter were transfected into HeLa cells with FuGene HD (Promega) according to the manufacturer’s protocol. Twenty-four hours after transfection, cells were treated with indicated amounts of dexamethasone, TNF-α, and/or vehicle. Twenty-four hours following treatment, firefly luciferase levels were measured as above. For figures, firefly divided by *Renilla* activity is shown, normalized to the control condition. GraphPad Prism was used to graph the data and statistical tests used are located in the figure legends.

The sequences cloned into the pGL3 reporter vectors are as follows: *IL8*, Chr4: 73,740,106-73,740,505; *ICAM1*, Chr19:10,270,782-10,271,081; *CCL2*, Chr17:34,252,540-34,252,896; and *RELB*, Chr19:45,001,228-45,001,545. All numbers correspond to the human GRCh38.p2 genome, accessed through *Ensembl*^65^. The *TSLP* construct has been reported previously^19^. Primers used to clone each of the above genomic regions are indicated in Supplementary Table 4.

### In vitro binding assays

Ten nM of double-stranded, 6-FAM-labeled DNA (Integrated DNA Technologies) was incubated with indicated amounts of protein in 100 mM NaCl, 20 mM Tris-HCl pH 7.4, and 5% glycerol. Formation of GR–DNA complexes was monitored via fluorescence polarization on a Biotek Synergy plate reader at an excitation and emission wavelength of 485 and 528 nm, respectively. Data were graphed and analyzed in Prism 6 (Graphpad Software). For GR DBD binding to κBREs, a two-site binding model was used.

Sequences of DNA constructs used for fluorescence polarization assays were: *PLAU*: 5′-(FAM) CCCTGGGAATTTCCTGATA-3′ and 5′-TATCAGGAAATTCCCAGGG-3′;

*CCL2*: 5′-GAGTGGGAATTTCCACTCA-3′ and 5′-TGAGTGGAAATTCCCACTC-3′;

*IL8*: 5′-(FAM) AATCGTGGAATTTCCTCTG-3′ and 5′-CAGAGGAAATTCCACGATT-3′. In all cases, (FAM) indicates the position of 6-FAM (fluorescein).

### Crystallization and structure analysis

GR DBD was concentrated to 3.0 mg/ml and complexed with DNA at a 1:1.2 molar ratio. Crystals of the GR DBD–*RELB* κBRE complex were grown in 0.1 M HEPES pH 7.5, 7 μM spermine, and 15% PEG 8000. Crystals of the GR DBD–*PLAU* κBRE complex were grown in 0.05 M sodium cacodylate, 0.05 M spermine, and 16% PEG 400. Crystals of the GR DBD–*CCL2* κBRE complex were grown in 0.1 M sodium malonate, 6% glycerol, and 5% PEG 3350. Crystals of the GR DBD–*IL8* κBRE were grown in 0.1 M HEPES pH 7.5, 7.5% glycerol, and 22% PEG 20000. Crystals of the GR DBD–*ICAM1* κBRE were grown in 0.1 M HEPES pH 7.7, 3% ethylene glycol, and 10% PEG 8000. DNA bases for figures are labeled by their position relative to the *RELB*-001, *PLAU*-001, *CCL2*-001, *CXCL8*-001, and *ICAM*-002 transcripts, respectively, accessed via *Ensembl*^65^. All crystals were grown at 20 °C via hanging drop vapor diffusion and flash cooled in mother liquor with the addition of 10–20% glycerol.

All X-ray data were collected at 1.00 Å wavelength at Southeast Regional Collaborative Access Team 22-ID and 22-BM beamlines at the Advanced Photon Source, Argonne National Laboratory. Supporting institutions may be found at www.ser-cat.org/members.html. Use of the Advanced Photon Source was supported by the U. S. Department of Energy, Office of Science, Office of Basic Energy Sciences, under Contract No. W-31-109-Eng-38. Structures were solved by molecular replacement using PHASER and refined with phenix.refine in the PHENIX suite^66^. Coot was used for visualization model rebuilding and PDB_REDO was used for validation and model improvement^67,68^. Figures were generated in MacPyMOL v1.7.0.3 (Schrödinger LLC). Sample electron density for all structures is reported in Supplementary Figure 5.

### NMR analysis

NMR data were collected on a Bruker 700 MHz (^1^H frequency) NMR instrument equipped with a QCI cryoprobe. For DNA NMR experiments, the 19 nt *IL8* κBRE DNA duplex was reconstituted in 20 mM phosphate (pH 6.7), 100 mM NaCl, 1 mM TCEP, 10% D_2_O buffer to a final concentration of 437 µM, subsequently annealed by denaturing at 95 °C for 3 min and equilibrated to room temperature (20–23 °C) overnight. A 2D ^1^H-detected NOESY was collected at 10 °C and 25 °C using 300 ms mixing time for *IL8* κBRE DNA before and after adding 0.44:1 or 2.3:1 of ^15^N-GR DBD. For protein NMR experiments, 2D [^1^H,^15^N]-HSQC spectra were collected at 25 °C for free ^15^N-GR DBD protein or protein complexed with 0.44:1 or 2.3:1 of *IL8* κBRE DNA duplex; or 1.5 × GBS consensus DNA sequence. Chemical shift perturbations were calculated using previously published GR DBD NMR chemical shifts^14^ and calculated using the minimum chemical shift perturbation procedure^69^ in the NMR analysis program NMRViewJ (OneMoon Scientific, Inc.).

### Sequence alignments

Sequences were retrieved from the Ensembl database^65^. Human sequences are from the GRCh38 genome build. Geneious version 6.1.6 (Biomatters Limited) was used for sequence alignment and visualization.

### Co-immunoprecipitation

WT and mutant GR were expressed in HEK293T cells (ATCC, CRL-3216) using the Tet-On^®^ Advanced inducible gene expression system (Takara Bio USA, Inc., Mountain View, CA). To this end, 10^6^ cells were seeded in each 6 cm dish, co-transfected the next day with the TransIT^®^-LT1 transfection reagent (Mirus Bio LLC, Madison, WI) and 2 μg/dish each of pTet-On Advanced reverse tetracycline-controlled transactivator (rtTA), pTight-FRT-Hygro2-HA-GR-WT/-S425G/- K442/R447A, and PCR3.1-p65-WT expression plasmids. Control cells were transfected with empty pTight vector instead of the GR expression plasmid. After 24 h, the media were replaced with phenol red-free DMEM + 10% csFBS, with or without 1 μg/ml Doxycycline. The next day, the cells were treated with 10 ng/ml TNF-α for 1 h and lysed in 600 μl RIPA buffer + protease inhibitor cocktail (P8340, Sigma-Aldrich Co. LLC, St. Louis, MO). Two hundred and fifty microliter aliquots of lysate were mixed with 1 μg of anti-HA (Y-11) antibody (Santa Cruz Biotechnology Inc., Dallas, TX) (Supplementary Table 3), 215 μl RIPA buffer, and 25 μl Dynabeads protein G (Invitrogen, Thermo Fisher Scientific Inc., Waltham, MA) and rotated overnight at 4 °C. The following day, the beads were washed 3 × with cold RIPA buffer and twice with cold phosphate-buffered saline (PBS). The beads were then incubated in 25 μl 2 × Laemmli sample buffer (1610737, Bio-rad Laboratories, Inc., Hercules, CA) for 5 min at 95 °C, and the supernatants were analyzed by western blotting using the anti-p65 (F-6) antibody (Supplementary Table 3).

### ChIP assay

Quantitative ChIP assay was performed as previously described^70^ with some modification. HEK293T transfectants in six-well plates and MCF-7 cells (ATCC) in 12-well plates were fixed in 11% formaldehyde for 15 min, quenched with 0.125 M glycine for 10 min, and rinsed with cold 1 × PBS. The cells were disrupted in lysis buffer^70^, incubated at 4 °C for 1 h and then sonicated. The lysates were then incubated with 100 μl Dynabeads protein G (Invitrogen, Thermo Fisher Scientific, Inc.) and 10 μl pre-immune rabbit IgG for 1 h at 4 °C, and centrifuged at 12,000 r.p.m. for 15 min at 4 °C. One hundred microliters of the pre-cleared supernatant was mixed with an antibody (Supplementary Table 3) (Santa Cruz Biotechnology, Inc.), 25 μl Dynabeads protein G (Invitrogen, Thermo Fisher Scientific Inc.), and lysis buffer to make a 200 μl IP mixture that were rotated overnight at 4 °C. The precipitates were sequentially washed in low-salt, high-salt, and LiCl buffers^70^, and twice in TE buffer. The crosslinks were then reversed at 65 °C for 3 h. DNA fragments were isolated using QIAquick PCR purification kit (QIAGEN, Hilden, Germany), and analyzed by qPCR using TaqMan^®^ 2 × master mix and custom TaqMan^®^ real-time PCR assays (Applied Biosystems, Thermo Fisher Scientific, Inc.) (Supplementary Table 4).

### Data availability

Coordinates and structure factors have been deposited in the Protein Data Bank under accession codes 5E69 [https://www.rcsb.org/structure/5E69], 5E6D[https://www.rcsb.org/structure/5E6D], 5E6A [https://www.rcsb.org/structure/5E6A], 5E6B[https://www.rcsb.org/structure/5E6B], and 5E6C [https://www.rcsb.org/structure/5E6C]. RNA-seq data have been deposited in the SRA under BioProject accession PRJNA314815 [https://www.ncbi.nlm.nih.gov/bioproject/?term=PRJNA314815]. Other data are available from the corresponding author upon reasonable request.

## Electronic supplementary material

Supplementary Information

Peer Review Report
