# Supplementary material for: Cryptic glucocorticoid receptor-binding sites pervade genomic NF-κB response elements
Source: Nat Commun. 2018 Apr 6;9:1337. doi: 10.1038/s41467-018-03780-1 (PMC5889392; doi:10.1038/s41467-018-03780-1)
Supplement: Supplementary file 1 — Supplementary Information [file 41467_2018_3780_MOESM1_ESM.pdf]

Supplementary Information for

Cryptic glucocorticoid receptor binding sites pervade genomic NF-κB response elements

Hudson et. al

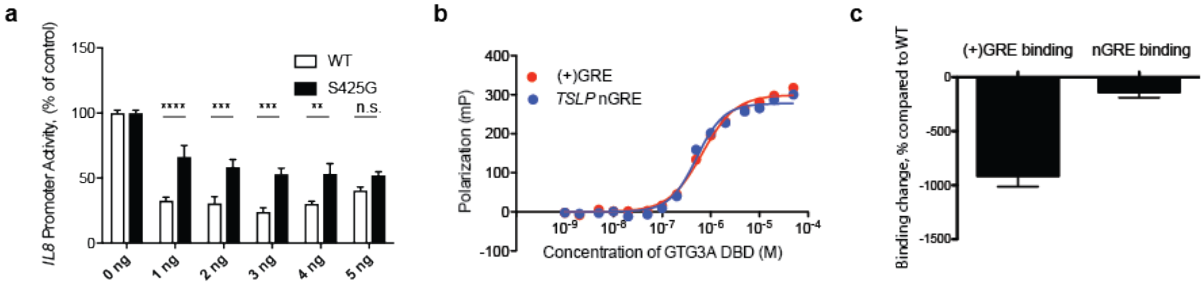

Supplementary Figure 1: GR DBD mutations have differential effects on the protein's DNA-binding subfunctions. (**a**) Full-length WT or S425G GR (in the pcDNA3.1 vector at the amount indicated) was transfected into HeLa cells, along with a constitutively-active *Renilla* luciferase gene as well as a firefly luciferase gene driven by the human *IL8* promoter flanked by *SV40* promoter and enhancer sequences. Reporter activity (mean ± s.e.m.) are from two independent experiments with at least two technical replicates at each plasmid concentration. (**b**) The GTG3A mutation[1] was tested for binding to a consensus (+)GRE and nGRE, in two independent replicates with three technical replicates each. Mean fluorescence polarization values are shown ± s.e.m. (**c**) Percentage change in affinity of the GTG3A mutant for (+)GREs and nGREs, compared to WT GR. The GTG3A mutant decreases the receptor's affinity for (+)GREs disproportionately, compared to nGREs.

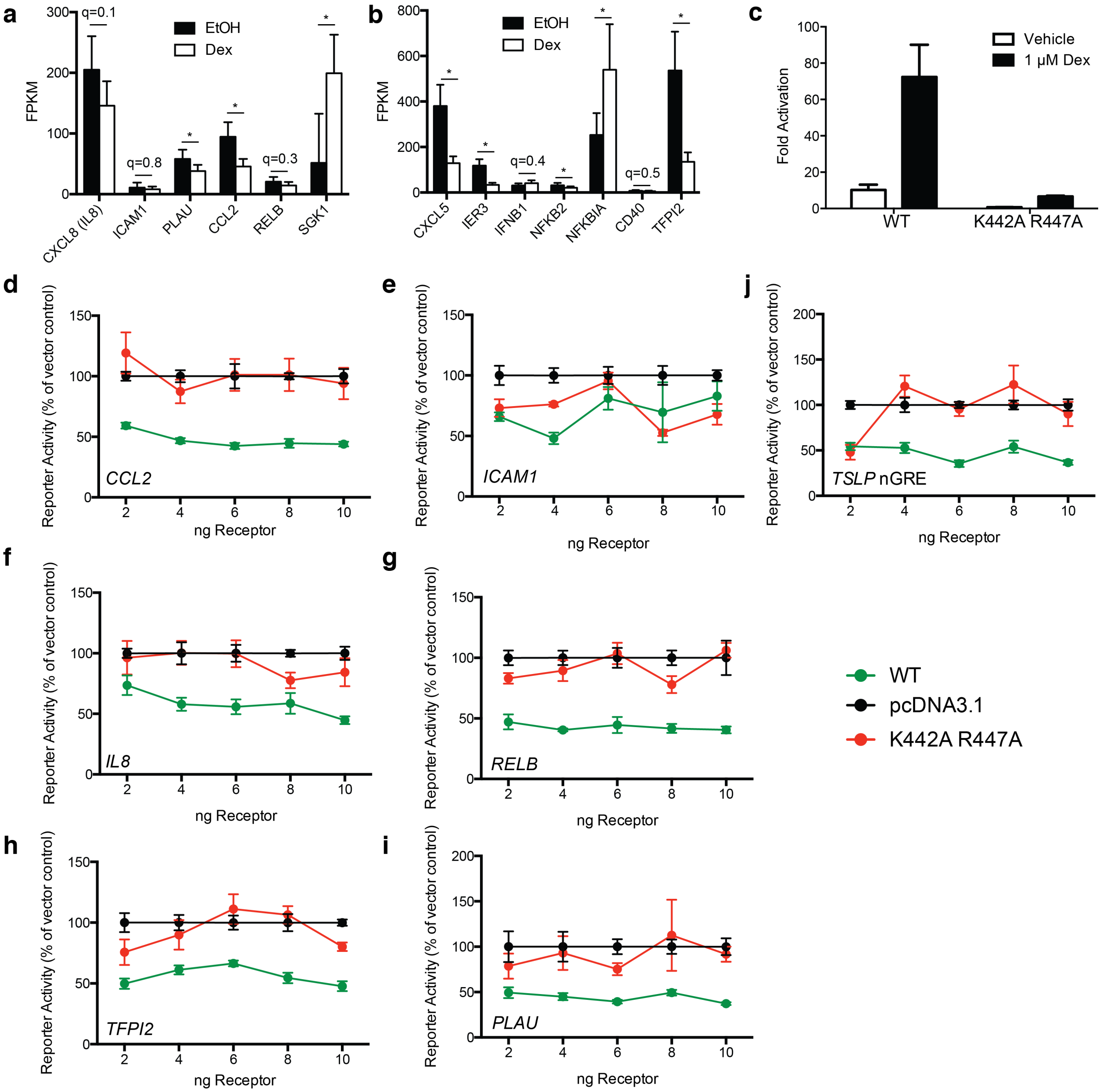

Supplementary Figure 2: The K442A R447A mutant is unable to repress constitutively active reporters containing κBREs. Dexamethasone treatment alone reduces the expression of many NF-κB regulated genes. (**a**) RNA-seq was performed on A549 cells treated with 100 nM dexamethasone or vehicle (see Methods for full details). Dexamethasone reduced the expression of all five genes whose promoters we crystallized in complex with the GR DBD, although the expression of *ICAM1* and *RELB* had low constitutive expression in A549 cells. *SGK1*, a (+)GRE-containing gene, is shown for comparison. (**b**) Additional genes with κBREs containing the AATTY consensus are also differentially expressed following dexamethasone treatment. (**c**) The K442A R447A mutant is greatly deficient in activation of the (+)GRE-containing *SGK* promoter in HeLa cells. (**d-i**) Increasing amounts of plasmid containing WT full-length GR, K442A R447A full-length GR, or empty pcDNA3.1 were transfected into HeLa cells containing the indicated κBRE constitutively activated by the SV40 promoter and enhancer. (**j**) The K442A

R447A mutant was also tested with the *TSLP* nGRE, which requires GR-DNA interaction for repression[2], as a control. All results are from at least two independent transfection experiments with four technical replicates each; mean and s.e.m. are shown.

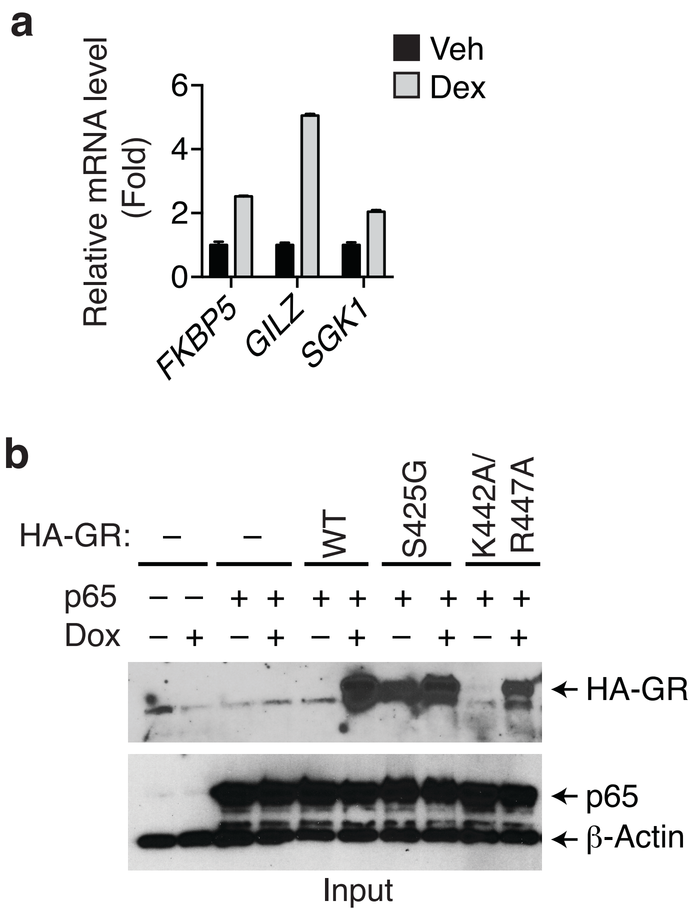

Supplementary Figure 3: Regulation of GR-target genes in HEK293T cells

(**a**) Dexamethasone up-regulates *GILZ, SGK1,* and *FKBP5* mRNA. Parental (i.e. un-transfected) HEK293T cells were incubated for 48 h in steroid-depleted media, and then stimulated with vehicle or 100 nM dexamethasone (Dex) for 12 h. Total RNA was extracted and analyzed by quantitative RT-PCR using inventoried TaqMan® gene expression assays (Applied Biosystems, Thermo Fisher Scientific Inc.) for *FKBP5* (Hs00296750_s1), *GILZ* (Hs00608272_m1), and *SGK1* (Hs00985033_g1). Expression levels (mean + s.e.m. n=2) are shown relative to *GAPDH* (4333764F). (**b**) Co-expression of GR and p65. HEK293T cells were transfected with p65 alone, or in combination with inducible, HA-tagged wild type and mutant GR plasmids, and treated with or without 1 μg/ml Dox as described in **Figure 2d**. Whole cell lysates were analyzed by western blot using antibodies against HA (Y-11), p65 (F-6), and β-actin (8H10D10).

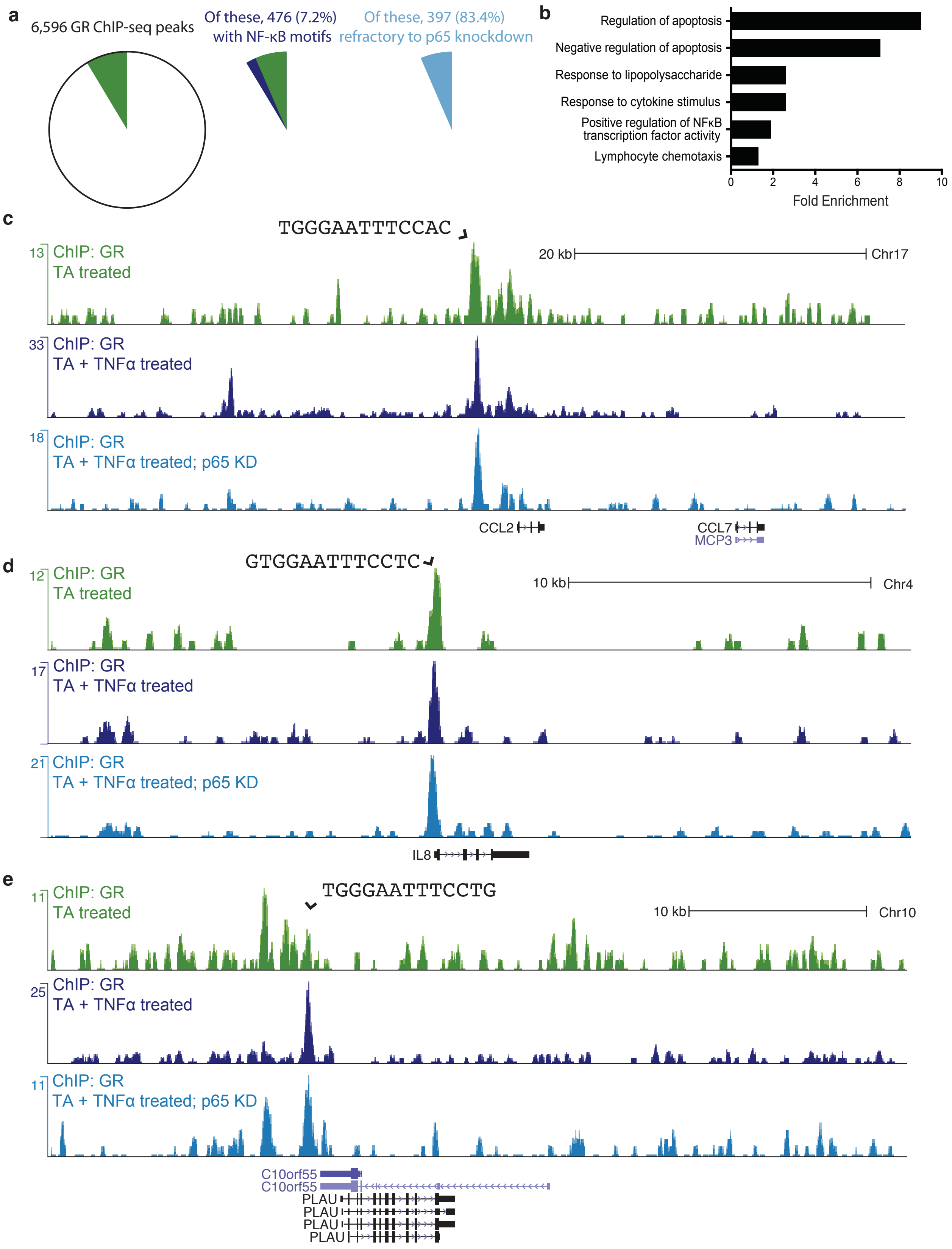
a
6,596 GR ChIP-seq peaks
Of these, 476 (7.2%) with NF-κB motifs
Of these, 397 (83.4%) refractory to p65 knockdown
b
Regulation of apoptosis
Negative regulation of apoptosis
Response to lipopolysaccharide
Response to cytokine stimulus
Positive regulation of NFκB transcription factor activity
Lymphocyte chemotaxis
0
2
4
6
8
10
Fold Enrichment
c
TGGGAATTTCCAC
20 kb
Chr17
13
ChIP: GR
TA treated
33
ChIP: GR
TA + TNFα treated
18
ChIP: GR
TA + TNFα treated; p65 KD
CCL2
CCL7
MCP3
d
GTGGAATTTCCTC
10 kb
Chr4
12
ChIP: GR
TA treated
17
ChIP: GR
TA + TNFα treated
21
ChIP: GR
TA + TNFα treated; p65 KD
IL8
e
TGGGAATTTCCTG
10 kb
Chr10
11
ChIP: GR
TA treated
25
ChIP: GR
TA + TNFα treated
11
ChIP: GR
TA + TNFα treated; p65 KD
C10orf55
C10orf55
PLAU
PLAU
PLAU
PLAU

Supplementary Figure 4: GR continues to occupy the *IL8*, *CCL2*, and *PLAU* κBREs after p65 knockdown. (**a**) Pie chart showing that of the 6,596 GR ChIP-seq peaks in HeLa cells treated with triamcinolone acetonide (TA) and without NF-κB activation, approximately 7% contain an κBRE. Of these peaks, the vast majority (83.4%) remains detectable when p65 is knocked down. (**b**) Selected enriched gene ontology terms for nearby genes to GR ChIP-seq peaks with κBREs that are refractory to p65 knockdown. All shown terms have a P value of < 0.05. (**c-e**) ChIP-seq tracks from Rao et al[3] of occupancy by GR after treatment with TA, TA + TNF-α, and TA + TNF-α with p65 knockdown on the *CCL2*, *IL8*, and *PLAU* κBREs (panels **c**, **d**, and **e**, respectively).

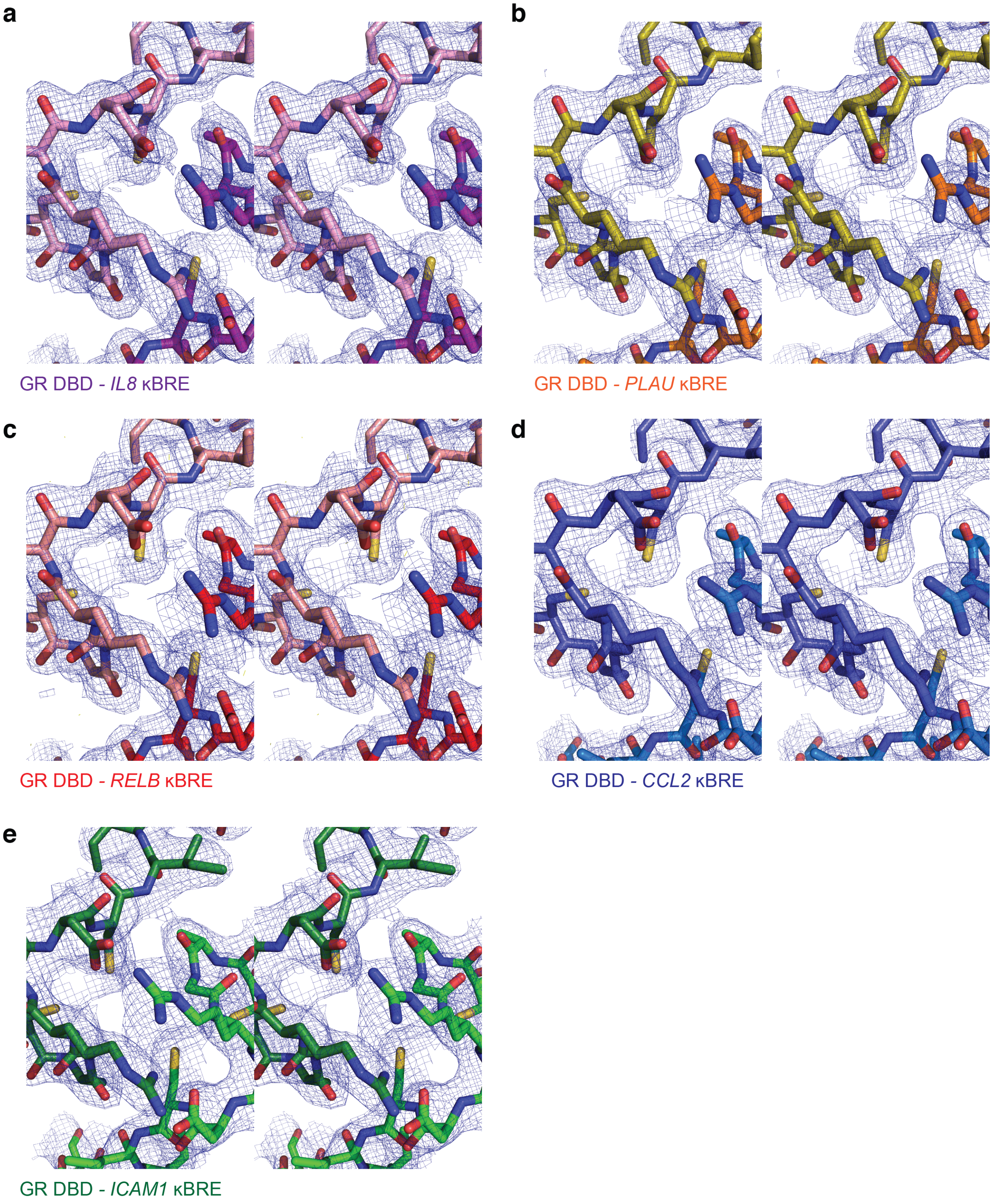

Supplementary Figure 5: Sample stereo electron density for all crystal structures reported in this article. $2F_o$-F_c maps at the interface between GR DBD monomers bound to the *IL8* (**a**), *PLAU* (**b**), *RELB* (**c**), *CCL2* (**d**), and *ICAM1* (**e**) κBREs. Separate protein chains are shown in different color shades. All maps are contoured to 1 σ.

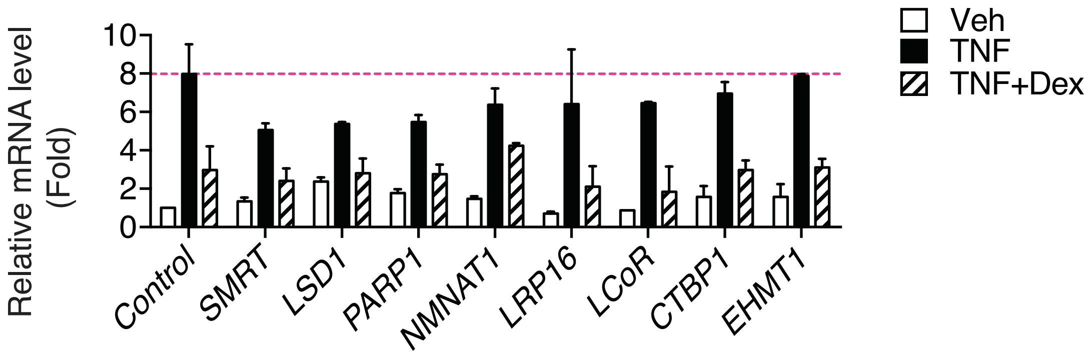

Supplementary Figure 6: Coregulator siRNAs that did not relieve *IL6* expression. MCF-7 cells transfected with control or the siRNAs against coregulators were stimulated with 10 ng/ml TNF-α alone or in combination with 10 nM dexamethasone for 2 hrs. Average *IL6* mRNA levels (mean ± s.e.m.; n = 3) determined by quantitative RT-PCR are shown relative to levels in control siRNA transfectants stimulated with the vehicle, which was arbitrarily set to 1.

Supplementary Figure 7. Uncropped images of gels and blots used in this study.

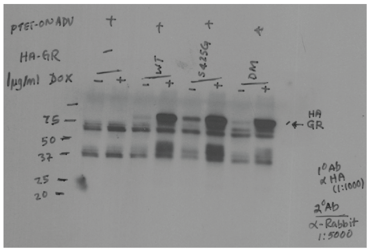

IB:
1°Ab: Anti-HA (Y-11)
Santa Cruz Biotech. sc-805
(1:1,000)
2° Ab: Goat anti-rabbit IgG-HRP
Cell Signaling Tech. #7074S
(1:5,000)

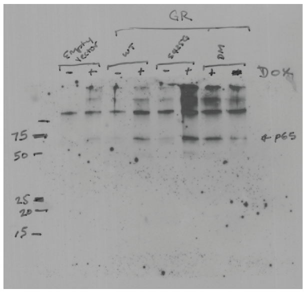

IP:
1 μg anti-HA (Y-11)
Santa Cruz Biotech. sc-805

IB:
1°Ab: Anti-p65 (F-6)
Santa Cruz Biotech. sc-8008
(1:1,000)
2° Ab: Goat anti-mouse IgG-HRP
Santa Cruz Biotech, sc-2005
(1:5,000)

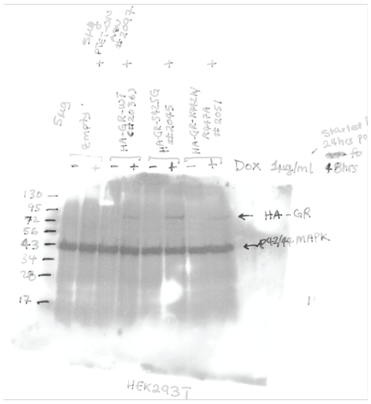

IB:
1°Ab: Anti-ERK1/2
Cell Signaling Tech. #4695S
(1:1,000)

2° Ab: Goat anti-rabbit IgG-HRP
Cell Signaling Tech. #7074S
(1:2,000)

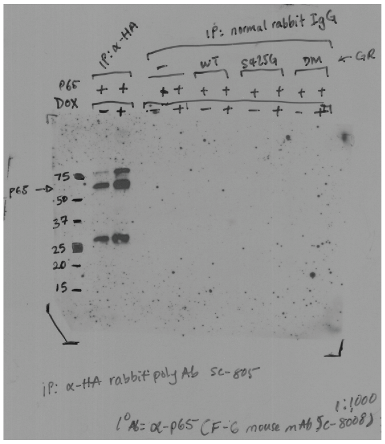

IP:
1 μg anti-HA (Y-11)
Santa Cruz Biotech. sc-805
or
1 μg normal rabbit IgG
Santa Cruz Biotech. sc-2027

IB:
1°Ab: Anti-p65 (F-6)
Santa Cruz Biotech. sc-8008
(1:1,000)
2° Ab: Goat anti-mouse IgG-HRP
Santa Cruz Biotech, sc-2005
(1:5,000)

Supplementary Table 1: Many glucocorticoid-regulated pro-inflammatory genes contain κBREs containing cryptic GR binding sites.

Gene	NF-κB response element sequence	Regulation by glucocorticoids	References
CARD15	GGG**AATTT**CC	Up	[5,6]
CCL2	GGG**AATTT**CC	Down	[7,8]
CCL5	AGA**AATTT**TTCC	Down	[9,10]
CD40	GGG**AATTT**CC	Down	[11,12]
CD40L	GG**AATTT**TCC	Up	[13,14]
CR2	GGG**AATTC**TCT	Up	[15,16]
CXCL5	GGG**AATTT**CCC	Down	[17,18]
ICAM1	GGA**AATTC**C	Down	[19]
IER3	CGG**AATTT**CC	Down	[8,20]
IFNB1	GGG**AAATT**CC	Down	[21,22]
IL8	GTGG**AATTT**CC	Down	[23]
NFKB2	GGG**AATTC**CC	Down	[8,24]
NFKBIA	GGA**AATTC**CCC	Down	[8,25]
PLAU	GGG**AATTT**CC	Down	[6,26]
RELB	GGGG**AATTC**C	Down	[8,27]
TFPI2	GGGG**AATTC**C	Down	[25,28]
TNC	GGG**AATTC**CT	Down	[29,30]

Supplementary Table 2: The cryptic GR binding footprint within κBREs is highly conserved. Shown are alignments for the four κBREs whose crystal structure in complex with GR DBD is reported here. The *IL8* alignment is shown in **Fig. 5b**. In each case, the GR binding footprint (bold) is more highly conserved than that of the flanking NF-κB footprint.

CCL2 κBRE
GR binding footprint:
92.9% identical
NF-κB binding footprint:
85.7% identical

homo_sapiens	CTGGG**AATTT**CCTGA
pan_troglodytes	CTGGG**AATTT**CCTGA
gorilla_gorilla	CTGGG**AATTT**CCTGA
pongo_abelii	CTGGG**AATTT**CCTGA
nomascus_leucogenys	CTGGG**AATTT**CCTGA
macaca_mulatta	CTGGG**AATTT**CCTGA
callithrix_jacchus	CTGGG**AATTT**CCTGG
microcebus_murinus	CTGGG**AATTT**CCTGA
otolemur_garnettii	CTGGG**AATTT**CCTGA
tupaia_belangeri	CTGGG**AATTT**CCTGA
cavia_porcellus	TGGGG**AATTT**CCTAA
dipodomys_ordii	TTGGG**AATTT**CCTAA
mus_musculus	CTGGG**AATTT**CCTAA
rattus_norvegicus	CTGGG**AATTT**CTTAA
ictidomys_tridecemlineatus	CTGGG**AATTT**CCTGA
ochotona_princeps	CTGGG**AATTT**CCTAA
oryctolagus_cuniculus	CTGGG**GATTT**CCTAA
ailuropoda_melanoleuca	CTGGG**AATTT**CCTGA
mustela_putorius_furo	CTGGG**AATTT**CCTGA
canis_familiaris	CTGGG**AATTT**CCTGA
felis_catus	CTGGG**AATTT**CCTGA
equus_caballus	CTGGG**AATTT**CCTGA
myotis_lucifugus	CTGGG**AATTT**CCTGA
pteropus_vampyrus	CCGGG**AATTT**CCTGA
bos_taurus	CTGGG**AATTT**CCTGA
tursiops_truncatus	CGGGG**AATTT**CCTGA
vicugna_pacos	CTGGG**AATTT**CCTGA
sus_scrofa	CTGGG**AAATT**CCTGA
erinaceus_europaeus	CTGGG**AAATT**CCTGA
sorex_araneus	CTGGG**AATTT**CCTGA
choloepus_hoffmanni	CGGGG**AATTT**CCTGA
dasypus_novemcinctus	CTGGG**AATTT**CCTGA
loxodonta_africana	CTGGG**AATTT**CCTGG
procavia_capensis	CTGGG**GATTT**CCAGA

ICAM1 κBRE
GR binding footprint:
100% identical
NF-κB binding footprint:
72.4% identical

homo_sapiens	CTTGG**AAATT**CCGG
pongo_abelii	CTTGG**AAATT**CCGG
nomascus_leucogenys	CTTGG**AAATT**CCGG
macaca_mulatta	CTCGG**AAATT**CCGG
microcebus_murinus	CTGGG**AAATT**CCGG
otolemur_garnettii	TCTGG**AAATT**CC--
cavia_porcellus	CTGGG**AAATT**CCGG
dipodomys_ordii	TTGGG**AAATT**CCGG
ictidomys_tridecemlineatus	TTTGG**AAATT**CCGA
ochotona_princeps	CTAGG**AAATT**CCGC
ailuropoda_melanoleuca	CTCGG**AAATT**CCAG
mustela_putorius_furo	GTCGG**AAATT**CCAG
felis_catus	TTTGG**AAATT**CCAG
myotis_lucifugus	TTCGG**AAATT**CCGG
pteropus_vampyrus	TTCTG**AAATT**CCGG
tursiops_truncatus	TTCGG**AAATT**CCGG
vicugna_pacos	TTCGG**AAATT**CCGG
echinops_telfairi	TTTGG**AAATT**CCGG
loxodonta_africana	CTTGG**AAATT**CGGG
procavia_capensis	ATCGG**AAATT**CCGA

RELB κBRE
GR binding footprint:
97.8% identical
NF-κB binding footprint:
93.0% identical

homo_sapiens	CCGGGG**AATTC**CGCCG
pan_troglodytes	CCGGGG**AATTC**CGCCG
gorilla_gorilla	CCGGGG**AATTC**CGCCG
pongo_abelii	CCGGGG**AATTC**CGCCG
callithrix_jacchus	CCGGGG**AATTC**CGCCG
otolemur_garnettii	CCGGGG**AATTC**CGCTG
tupaia_belangeri	CCGCTC**TATTC**CGCCG
cavia_porcellus	CCGGGG**AATTC**CGCCG
dipodomys_ordii	CCGGGG**AATTC**CGCCG
mus_musculus	CCGGGG**AATTC**CGCCG
rattus_norvegicus	CCGGGG**AATTC**CGCCG
ictidomys_tridecemlineatus	CCGGGG**AATTC**CGCCG
canis_familiaris	CCGGGG**AATTC**CGCTG
felis_catus	CCGGGG**AATTC**CGCCG

PLAU κBRE
GR binding footprint:
95.4% identical
NF-κB binding footprint:
91.4% identical

homo_sapiens	CTGGG**AATTT**CCTGA
pan_troglodytes	CTGGG**AATTT**CCTGA
gorilla_gorilla	CTGGG**AATTT**CCTGA
pongo_abelii	CTGGG**AATTT**CCTGA
nomascus_leucogenys	CTGGG**AATTT**CCTGA
macaca_mulatta	CTGGG**AATTT**CCTGA
callithrix_jacchus	CTGGG**AATTT**CCTGG
microcebus_murinus	CTGGG**AATTT**CCTGA
otolemur_garnettii	CTGGG**AATTT**CCTGA
tupaia_belangeri	CTGGG**AATTT**CCTGA
cavia_porcellus	TGGGG**AATTT**CCTAA
dipodomys_ordii	TTGGG**AATTT**CCTAA
mus_musculus	CTGGG**AATTT**CCTAA
rattus_norvegicus	CTGGG**AATTT**CTTAA

pteropus_vampyrus	CCGGGG**AATTC**CGCCG	ictidomys_tridecemlineatus	CTGGG**AATTT**CCTGA
bos_taurus	CCGGGG**AATTC**CGCCG	ochotona_princeps	CTGGG**AATTT**CCTAA
loxodonta_africana	CCGGGG**AATTC**CGCTG	oryctolagus_cuniculus	CTGGG**GATTT**CCTAA
procavia_capensis	CCGGGG**AATTC**CGCTG	ailuropoda_melanoleuca	CTGGG**AATTT**CCTGA
		mustela_putorius_furo	CTGGG**AATTT**CCTGA
		canis_familiaris	CTGGG**AATTT**CCTGA
		felis_catus	CTGGG**AATTT**CCTGA
		equus_caballus	CTGGG**AATTT**CCTGA
		myotis_lucifugus	CTGGG**AATTT**CCTGA
		pteropus_vampyrus	CCGGG**AATTT**CCTGA
		bos_taurus	CTGGG**AATTT**CCTGA
		tursiops_truncatus	CGGGG**AATTT**CCTGA
		vicugna_pacos	CTGGG**AATTT**CCTGA
		sus_scrofa	CTGGG**AAATT**CCTGA
		erinaceus_europaeus	CTGGG**AAATT**CCTGA
		sorex_araneus	CTGGG**AATTT**CCTGA
		choloepus_hoffmanni	CGGGG**AATTT**CCTGA
		dasypus_novemcinctus	CTGGG**AATTT**CCTGA
		loxodonta_africana	CTGGG**AATTT**CCTGG
		procavia_capensis	CTGGG**GATTT**CCAGA

Supplementary Table 3. List of antibodies

Antibody		Source	Catalog No.
HA	Y-11	Santa Cruz Biotechnology Inc.	sc-805
β-actin	8H10D10	Cell Signaling Technology	3700
ERK1/2	137F5	Cell Signaling Technology	4695
GR	H-300	Santa Cruz Biotechnology Inc.	sc-8992
ERα	HC-20	Santa Cruz Biotechnology Inc.	sc-543
p65 (ChIP)	C-20	Santa Cruz Biotechnology Inc.	sc-372
p65 (WB)	F-6	Santa Cruz Biotechnology Inc.	sc-8008
c-Jun	H-79	Santa Cruz Biotechnology Inc.	sc-1694
JunB	N-17	Santa Cruz Biotechnology Inc.	sc-46
JunD	329	Santa Cruz Biotechnology Inc.	sc-74
c-Fos	K-25	Santa Cruz Biotechnology Inc.	sc-253
FosB	H-75	Santa Cruz Biotechnology Inc.	sc-7203
Fra-1	H-50	Santa Cruz Biotechnology Inc.	sc-22794
Fra-2	H-103	Santa Cruz Biotechnology Inc.	sc-13017
HDAC1	H-51	Santa Cruz Biotechnology Inc.	sc-7872
SRC2/Grip-1	R-91	Santa Cruz Biotechnology Inc.	sc-28934
Normal rabbit IgG		Santa Cruz Biotechnology Inc.	sc-2027

Supplementary Table 4. Primers used

Promoter	Forward (5'–3')	Reverse (5'–3')	Probe (FAM-5'–3'-MGB)
**Custom Taqman® real-time PCR assays**			
IL6	CCCTCACCCTCCAACAAAGATTTAT	GCCTCAGACATCTCCAGTCCTATAT	AAATGTGGGATTTTCC
IL8	GAAGTGTGATGACTCAGGTTTGC	TGCACCCTCATCTTTTCATTATGTCA	CAAATCGTGGAATTTC
ICAM1	AGGGAGGACTTGAGTTCGGA	AATACTGCCAACTTCCCCGG	TGGAGTCTCAGTTTACCGCT
GILZ	CCGTTGCTGCTCTGCTATTG	TTCCCTGTCAGAGCAAGCAC	GCTGTTGCCAGACATCCAAT
SGK1	TGTCAGCGTCCATCCAAATG	ACAGCATGATTGATCCTCAGC	TGGGCACAGTGAGATGACTC
FKBP5	CGAGCTGCAAAACATCACTT	AGGGTGTTCTGTGCTCTTCAA	CTGCCCTAGAGCAATTTTGTT
**Custom Primers for Cloning LUC vectors**			
IL8	ATTTATTTTAAATTACCTCCCCAATAAAATG	GAATTTCCTCTGACATAATGAAAAGAT	
ICAM	GAAGTTGGCAGTATTTAAAAGTACTTAA	CACCTGGGGGCCAA	
CCL2	GCTTGTGCCGAGATGTTCCC	CATGTCTCTACTTCAGGAAGG	
RELB	CAGTTGTCTCGTCCAGAGCAATG	TCTGGTGGACGATCGCG	

Supplementary Table 5: Fluorescence polarization was used to monitor binding of GR DBD to four human κBREs. These binding events showed two-site binding curves, similar to that of GR DBD when binding to an nGRE[4]. An extra sum-of-squares F-test was used to compare a two-site specific binding event to a one-site specific binding event; the resulting *p* values are shown in the left column of the panel.

Response element	High site K_d	Low site K_d	p for 2-site binding
IL8	239 nM	17.0 μM	> 0.0001
IL6	221 nM	68.5 μM	> 0.0001
CCL2	233 nM	67.9 μM	> 0.0001
PLAU	215 nM	>50 μM	0.0051

Supplementary References

1. Caldenhoven, E. et al. Negative cross-talk between RelA and the glucocorticoid receptor: a possible mechanism for the antiinflammatory action of glucocorticoids. *Mol Endocrinol* **9**, 401-12 (1995).
2. Surjit, M. et al. Widespread negative response elements mediate direct repression by agonist-liganded glucocorticoid receptor. *Cell* **145**, 224-241 (2011).
3. Rao, N.A. et al. Coactivation of GR and NFKB alters the repertoire of their binding sites and target genes. *Genome Res* **21**, 1404-16 (2011).
4. Hudson, W.H., Youn, C. & Ortlund, E.A. The structural basis of direct glucocorticoid-mediated transrepression. *Nat Struct Mol Biol* **20**, 53-58 (2012).
5. Gutierrez, O. et al. Induction of Nod2 in Myelomonocytic and Intestinal Epithelial Cells via Nuclear Factor kappaB Activation. *J Biol Chem* **277**, 41701-41705 (2002).
6. Lu , N.Z., Collins, J.B., Grissom, S.F. & Cidlowski, J.A. Selective Regulation of Bone Cell Apoptosis by Translational Isoforms of the Glucocorticoid Receptor. *Mol Cell Biol* **27**, 7143-7160 (2007).
7. Ueda, A. et al. NF-kappa B and Sp1 regulate transcription of the human monocyte chemoattractant protein-1 gene. *J Immunol* **153**, 2052-63 (1994).
8. Rao, N.A.S. et al. Coactivation of GR and NFKB alters the repertoire of their binding sites and target genes. *Genome Res* **21**, 1404-1416 (2011).
9. Moriuchi, H., Moriuchi, M. & Fauci, A.S. Nuclear factor-kappa B potently up-regulates the promoter activity of RANTES, a chemokine that blocks HIV infection. *J Immunol* **158**, 3483-91 (1997).
10. Castro, M. et al. Asthma Exacerbations after Glucocorticoid Withdrawal Reflects T Cell Recruitment to the Airway. *Am J Respir Crit Care Med* **169**, 842-849 (2004).
11. Hinz, M. et al. Constitutive NF-kappaB maintains high expression of a characteristic gene network, including CD40, CD86, and a set of antiapoptotic genes in Hodgkin/Reed-Sternberg cells. *Blood* **97**, 2798-2807 (2001).
12. Piemonti, L. et al. Glucocorticoids Affect Human Dendritic Cell Differentiation and Maturation. *J Immunol* **162**, 6473-6481 (1999).
13. Schubert, L.A. et al. A T Cell-specific Enhancer of the Human CD40 Ligand Gene. *J Biol Chem* **277**, 7386-7395 (2002).
14. Jabara, H.H., Brodeur, S.R. & Geha, R.S. Glucocorticoids upregulate CD40 ligand expression and induce CD40L-dependent immunoglobulin isotype switching. *J Clin Invest* **107**, 371-378 (2001).
15. Tolnay, M., Vereshchagina, L.A. & Tsokos, G.C. NF-kappaB Regulates the Expression of the Human Complement Receptor 2 Gene. *J Immunol* **169**, 6236-6243 (2002).
16. Galon, J. et al. Gene profiling reveals unknown enhancing and suppressive actions of glucocorticoids on immune cells. *FASEB J* **16**, 61-71 (2002).
17. Keates, A.C. et al. ZBP-89, Sp1, and Nuclear Factor-kappaB Regulate Epithelial Neutrophil-activating Peptide-78 Gene Expression in Caco-2 Human Colonic Epithelial Cells. *J Biol Chem* **276**, 43713-43722 (2001).
18. Ishmael, F.T. et al. Role of the RNA-Binding Protein Tristetraprolin in Glucocorticoid-Mediated Gene Regulation. *J Immunol* **180**, 8342-8353 (2008).

19. Tanaka, Y., Hayashi, M., Takagi, S. & Yoshie, O. Differential transactivation of the intercellular adhesion molecule 1 gene promoter by Tax1 and Tax2 of human T-cell leukemia viruses. *J Virol* **70**, 8508-17 (1996).
20. Huang, Y.-H., Wu, J.Y., Zhang, Y. & Wu, M.X. Synergistic and opposing regulation of the stress-responsive gene IEX-1 by p53, c-Myc, and multiple NF-kappaB/rel complexes. *Oncogene* **21**, 6819-6828 (2002).
21. Hiscott, J. et al. Induction of human interferon gene expression is associated with a nuclear factor that interacts with the NF-kappa B site of the human immunodeficiency virus enhancer. *J Virol* **63**, 2557-2566 (1989).
22. Baudy, A.R. et al. delta9,11 Modification of Glucocorticoids Dissociates Nuclear Factor-kappaB Inhibitory Efficacy from Glucocorticoid Response Element-Associated Side Effects. *J Pharmacol Exp Ther* **343**, 225-232 (2012).
23. Kunsch, C., Lang, R.K., Rosen, C.A. & Shannon, M.F. Synergistic transcriptional activation of the IL-8 gene by NF-kappa B p65 (RelA) and NF-IL-6. *J Immunol* **153**, 153-64 (1994).
24. Lombardi, L. et al. Structural and functional characterization of the promoter regions of the NFKB2 gene. *Nucleic Acids Res* **23**, 2328-2336 (1995).
25. Hinz, M. et al. Nuclear factor kappaB-dependent gene expression profiling of Hodgkin's disease tumor cells, pathogenetic significance, and link to constitutive signal transducer and activator of transcription 5a activity. *J Exp Med* **196**, 605-617 (2002).
26. Wang, Y. et al. Identification of a novel nuclear factor-kappaB sequence involved in expression of urokinase-type plasminogen activator receptor. *Eur J Biochem* **267**, 3248-3254 (2000).
27. Bren, G.D. et al. Transcription of the RelB gene is regulated by NF-kappaB. *Oncogene* **20**, 7722-7733 (2001).
28. Haupl, T. et al. Gene Expression Profiling of Rheumatoid Arthritis Synovial Cells Treated with Antirheumatic Drugs. *J Biomol Screen* (2007).
29. Mettouchi, A. et al. The c-Jun-induced transformation process involves complex regulation of tenascin-C expression. *Mol Cell Biol* **17**, 3202-9 (1997).
30. Grundberg, E. et al. Global Analysis of the Impact of Environmental Perturbation on cis-Regulation of Gene Expression. *PLoS Genet* **7**, e1001279 (2011).
