# Supplementary material for: Cryptic glucocorticoid receptor-binding sites pervade genomic NF-κB response elements
Source: Nat Commun. 2018 Apr 6;9:1337. doi: 10.1038/s41467-018-03780-1 (PMC5889392; doi:10.1038/s41467-018-03780-1)
Supplement: Supplementary file 2 — Peer Review Report [file 41467_2018_3780_MOESM2_ESM.pdf]

Reviewers' comments:

Reviewer #1 (Remarks to the Author):

Glucocorticoids are important drugs in anti-inflammatory treatments. It is well known that this involves both the glucocorticoid receptor (GR) and the transcription factor NF-κB, but the detailed mechanism of action is not known. Currently the most popular hypothesis is based on a direct interaction between activated GR and NF-κB (the "tethering" hypothesis).
In this manuscript, however, the authors provide strong evidence for an alternative mechanism involving binding of GR to DNA sequences within the NF-κB response elements (κBREs). They discovered that GR binds to κBREs at highly conserved sequences AATTY (where Y is a pyrimidine) both in vitro and in vivo. They further characterized GR-DNA binding with X-ray crystallography of GR-DNA complexes and by NMR spectroscopy. The results from a combination of structural and other methods (mutagenesis, Chip, fluorescence polarization, etc.) make a very strong case for the sequence-specific DNA-binding mechanism.
The paper is novel and interesting. The results are very well documented and important for the inflammation field. I have only a few minor suggestions to improve the readability for non-experts:
- As the authors have used many abbreviations (not all well-defined like LPS on p.1) I would suggest to include a glossary of abbreviations in the main text.
- The use of a superscript for a reference in combination with a number or formula may be confusing. It can be replaced by "(ref. 27)" or equivalent.
With these changes the manuscript can be accepted for publication in Nature Com.

Reviewer #2 (Remarks to the Author):

In this manuscript Ortlund and colleagues describe the identification of novel binding sites for the DNA binding domain (DBD) of the glucocorticoid receptor. This is the latest contribution to a larger body of work from this lab attempting to map the GR DBD binding sites and their associated functions seeking to define a novel paradigm for GR action which moves past the time honored but imperfect activation vs repression paradigm for GR. In the opinion of this reviewer the work represents another chapter in understanding the role of NFKB binding sites in GR signaling. Yet the impact of the conclusions of this paper is not substantial enough to warrant publication in Nature Communications. The authors could consider some suggestions below to add impact, or if they wish, the contents of this manuscript should definitely be published in another journal.
The science is excellent. The authors show a deep understanding of GR and its associated signaling / binding sites. The identification of the kBREs is notable, and the crystallography experiments are well done. The NMR work clearly supports the X ray data regarding the kBRE binding. Yet it seems the previous publication in Nature Structural and Molecular Biology in 2013 where the nGRE sites were similarly identified led to a very similar set of experiments examining a subset of the kBREs discussed here. Many of the figures and tables follow the example of this earlier publication.The authors do demonstrate that the kBREs investigated are not 'tethered' to the DNA sequence rather a direct interaction has been noted for these response elements. The direct binding of the GR mutants studied is observed for SOME kBRE containing promoters but the authors have carefully selected them to avoid effects by stimuli (TNF) or glucocorticoids (dexamethasone, triamcinolone acetonide) despite the statement that a large number of these genes are affected by both agents with many under kBRE control. This is perhaps of greatest concern: Identification of these sites selectively and defining the associated crystal structure is impressive yet any association of these binding sites with the stimuli or steroid would be highly impactful. Why for example would one be interested in GR binding to any sequence of DNA in the absence of ligand? Is there a novel constitutive component of GR activity that is not characterized in the isoforms of this receptor with a missing LBD? Steps towards resolution of ligand, stimulus, and function at the level of

transcription are prized goals for nuclear hormone research. The authors have mentioned so called differentiated ligands in their body of work that have been examined for decades. Would it be worthwhile to characterize one of these (compound A?) as a start towards understanding the gene response of non steroidal ligands showing differentiated function? Efforts to this end would add impact and be highly attractive to the readers of Nature Communications.
All of that said the body of work from these authors has been impressive. Their efforts have provided a novel paradigm for GR monomeric vs dimeric gene regulation that could lead to a novel paradigm for GR modulation by NCEs. This manuscript offers another short chapter in the characterization of one such response element associated with some NFKB responsive genes and as such adds to the impressive story of this investigation. However the impact of this particular chapter is eclipsed by earlier publications where the process, results and expectations were similarly described for another DNA response element. Hence my recommendation to enhance this work or publish elsewhere.
I have enclosed a proof written version of the original submission for the benefit of the authors. Perusal of this annotated pdf should assist the needed revisions and point out simple corrections. The authors are encouraged to check their reference management software as there are many duplicated citations in the reference section (12 & 65, 19&22, 29 & 30).

Reviewer #3 (Remarks to the Author):

The team of Eric Ortlund reports on the presence of cryptic GR binding sites positioned at genomic NF-kappaB binding sites, and proposes this mechanism as a likely contributor to the repression of inflammatory genes by glucocorticoids. In line with earlier findings of Uhlenhaut et al., who reported on tethering being unlikely as a mechanism to explain GR-induced gene repression mechanisms at inflammatory gene promoters, the current study has aimed to moved further and to propose how this alternative mechanism may work. Regardless of how attractive and plausible this novel hypothesis may be, the molecular biology part of the study unfortunately has several shortcomings, with data presented in an unclear manner and/or missing a fair amount of control experiments to scientifically back up the statements, and to unambiguously draw solid conclusions. In many experiments, statistical analyses are lacking.

Major comments
1) An important comment is why authors have used throughout their study, for many experiments only GR-DBD while they clearly show that they also have a baculovirus-expressed full length GR at over 99% purity. To strengthen their statements and hypothesis, authors should be able to recapitulate their findings with this full length GR, instead of with a partial GR moiety, which may not reflect a physiologically relevant situation.
2) A second important comment is that authors should back up their findings by including a gelshift analysis, using both unliganded versus liganded recombinant FL GR, as well as a control versus dex-activated cell lysate, on these isolated cryptic GR binding NFkappaB elements. Supershift analysis using an antibody against GR should demonstrate GR presence.
3) Could GR bind to these cryptic sites in absence of NFkappaB? In other words, can a mutant site be identified that eradicates NFkappaB binding, yet retains GR binding?
4) Around the world, in different labs, many commercially available NF-kappaB promoters linked to luciferase are being used. Have authors checked whether these also contain the cryptic GR binding sites?
5) P3 and Fig S1a. The authors claim that GR Ser425Gly, a mutant that fails to bind DNA and to interact with NFkappaB, is unable to repress the constitutively active IL-8 promoter.
a) I fail to see this: in Fig S1a, as compared to control levels, there is indeed a larger difference as compared to GR wildtype, yet a mild, yet consistent, inhibitory response is visible also for GR S425G? Please clarify.

b) Transfection procedures may also cause a minimal activation of NFkappaB, and the mutant may rather be compromising NFkappaB binding instead of killing the interaction completely, which might explain residual inhibitory effects. Authors should check whether mutating the NFkappaB binding site, would still yield suppression or not with both constructs.
c) Statistical analysis is lacking for control versus transfected levels with S425, or wt, which makes the conclusion anyway uncertain. Number of independent experiments is missing.
d) If statistics would reveal there is indeed a residual inhibitory effect, then what could be the explanation (in case b) could be ruled out)? Can the presence of other TF binding sites that may still be present in this promoter, explaining this remaining ability to repress?
e) This experiment further lacks an essential control: a Western analysis of transfected protein levels for both GR wt and GR S425G and a loading control should accompany this result.
f) Further, the authors have made the 400bp IL-8 reporter “constitutively” active by flanking a constitutively active viral minimal promoter, but it was not shown whether or not this construct remains functional and still behaves as expected when induced with an inflammatory signal, IL-1beta or TNF.
g) It is not stated in the methods whether or not for this experiment, the total amount of transfected DNA has been kept constant (only for FigS2).
6) Fig. S1b and c: statistical analysis is missing. N=?
7) Fig S1b and c: authors should explain the implication of these results in more detail in the results section, for the separate graphs. S1c is difficult to interpret, it would be easier to interpret and allowing direct comparison, when results for GRwt are also depicted in FigS1b.
8) Fig S1c: the Y-axis states “% compared to wt”, but the numbers on the graph do not seem to indicate this.
9) P3. The results section mentions data on 2 GR mutants, A458T and GTG3A, yet the referencing is unclear and the graph only shows results with GTG3A. Are data in the graph missing?
10) P3. The final conclusion from FigureS2 is not novel and already supported in 1997 by the work of Lidén et al, as well as of Wissink et al.
11) Figure 1 (c-e). Authors rely on results of another study. How efficient was the knockdown of p65 (this should at least be stated)? Lack of signal for p65, at sites where a signal for GR is present, could have multiple reasons, e.g. the remainder p65 epitope could be masked (as could be expected when a tethering takes place), or either for the depicted sites the NFkappaB binding site could be occupied by other NFkappaB family members. The discussion on this should therefore be better nuanced.
12) Figure 1f. Please indicate in what region the ChIP primers were chosen, and where the respective TF binding sites are localized.
13) Figure 1f. Is this performed in HEK293T (which contain very little GR basally, and are therefore often used as a cell line to check GR mutant functionality) or on GR-transfected HEK293T? This is unclear. Authors state further that there are no potential tethering factors present, yet a signal for Fra 1-2 is shown, so please clarify why this is disregarded?
14) Figure 1f. Authors must also realize that in the IL-6 promoter other TF binding sites are present which could contribute, eg. CREB (which is also a potential GR-tethering factor).
15) Figure 1g. the most important control is missing and this is p65.
16) Figure 2d. Corresponding controls for this experiment are missing, i.e. the loading control for p65 overexpression and also the negative antibody control for the immunoprecipitation (IgG control). Plus, the loading control for HA-GR is also missing and it cannot be extrapolated from Figure 2a, since this is another experiment (inductions at different times and in absence of p65 overexpression).
17) Figure 2b. What is the % recruitment over input?
18) Figure 2b. A negative control of recruitment levels in absence of overexpressed GR is missing. Also, why is there a strong signal of HA-GR recruitment for GILZ, SGK1 and FKBP5 in absence of ligand? Is this the consequence of the overexpression? Connected herewith, in Figure S5, how do basal mRNA compare, i.e. are these genes also basally activated in absence of DEX? Plus, in Figure S5, is HA-GR here actually overexpressed or not? (HEK293T, low in endogenous GR, are in other experiments always supplemented with GR).
19) Figure 2b. Why is the signal consistently lower for recruitment of HA-GR at IL-6, ICAM and IL-

8 promoters, upon activation of GR with DEX (third lanes vs. second lanes). This would almost indicate that a ligand-independent binding event is being favored at these sites? Please clarify.
20) Figure 2b. Recruitment of HA-GR at the IL-6 promoter with GR S425G. Why is the third lane of the hatched bars missing and hence, why is this signal not consistent with the other promoters?
21) Figure 2b, pertaining to the four questions above, statistics are missing (n=3 so it should be possible to do this analysis at an averaged figure result) so it is difficult if not impossible to draw definite conclusions from these results.
22) Figure 2b, assuming changes are statistically significant, how do authors explain that the highest recruitment of HA-GR at IL-6, ICAM and IL8 promoters in presence of DEX/TNFalpha. Can a TNF-induced modification lie at the basis, or can a classic tethering mechanism
23) Figure 6d. Y-axis is unclear, what is measured? Luciferase? Not sure conclusions are valid here, as data can also oppositely be interpreted that the TNF-inducing ability is lower for the mutated sites, without affecting the gene repressive capacity of dex-activated GR.
24) Figure S4d: have authors checked the binding behavior of the reverse control experiment, i.e. p65 DBD in this assay?
25) In the discussion section, p8 second paragraph, authors list several mechanisms that do not have to be exclusive but may co-exist with the novel reported mechanism. They state that ChIPseq studies have not yet validated a widespread occupancy of IR-GBSs by GR, potentially due to a lower affinity for GR compared to dimeric GBS elements. Surely, the same argument would also be the case for protein-protein interactions, as in the case of the classic tethering model, and a failure to find support for this model by a classic ChIP approach ChIP could, by extension, also be caused by variation in experimental ChIPping conditions, as it is more difficult to trap these interaction types as opposed to a direct protein-DNA interaction. This argument should also be integrated in the discussion.

Minor comments
1) Overall, Figures are not sufficiently self-explanatory and legends are therefore rather extensive.
2) In the abstract authors state the cryptic sites are recognized between the binding sites of NF-kappaB, but this may confusingly also be interpreted as “besides” multiple NFkappaB binding sites in a given promoter, and not at the actual NFkappaB binding site. Please revise.
3) Although the PDB code is given in the legend, in Fig 1B. it could be just defined from which gene promoter the canonical GBS originates.
4) Ref22 should be deleted as it is the same as ref 19.
5) Fig S1a. How are graph results calculated: if % of control response, should the first bars not be set at 100?
6) Figure 1 (c-e), in the legend ref 25 is wrongly cited. Please check.
7) Figure 1 (c-e). For the read in presence of activated p65, i.e. the controls, where do the p65 peaks localize? Including these results would be helpful.
8) Figure 1f. “in some instances”. Please rephrase, for the sake of clarity.
9) Figures 1f and g are both ChIP results, why are different Y-axis used? ChIP binding results should be depicted as %binding over input control.
10) Figure S4 seems as a key result. Why do authors present this as Supplementary data?
11) P 8. First paragraph, last sentence. Part of the sentence is missing to be understandable. Please fix.
12) Figure 5e. Define green in legend.
13) Figure 6c. What assay has been done here? Please specify.
14) Ref 29 and 30 are duplicates.

Reviewer #1 (Remarks to the Author):

Glucocorticoids are important drugs in anti-inflammatory treatments. It is well known that this involves both the glucocorticoid receptor (GR) and the transcription factor NF-κB, but the detailed mechanism of action is not known. Currently the most popular hypothesis is based on a direct interaction between activated GR and NF-κB (the "tethering" hypothesis).

In this manuscript, however, the authors provide strong evidence for an alternative mechanism involving binding of GR to DNA sequences within the NF-κB response elements (κBREs). They discovered that GR binds to κBREs at highly conserved sequences AATTY (where Y is a pyrimidine) both in vitro and in vivo. They further characterized GR-DNA binding with X-ray crystallography of GR-DNA complexes and by NMR spectroscopy. The results from a combination of structural and other methods (mutagenesis, Chip, fluorescence polarization, etc.) make a very strong case for the sequence-specific DNA-binding mechanism.

The paper is novel and interesting. The results are very well documented and important for the inflammation field. I have only a few minor suggestions to improve the readability for non-experts:

We thank the reviewer for these comments and have addressed his/her suggestions below:

- As the authors have used many abbreviations (not all well-defined like LPS on p.1) I would suggest to include a glossary of abbreviations in the main text.

We updated the main text where abbreviations were not properly introduced (e.g. LPS, ChIP, for example) and are happy to add a table of abbreviations to the end of the main text if this is in line with the NCOMMS policy.

- The use of a superscript for a reference in combination with a number or formula may be confusing. It can be replaced by "(ref. 27)" or equivalent.

We agree. We have added "(ref. x)" where necessary to avoid a superscript occurring after a number on page 6, 10, and 11.

With these changes the manuscript can be accepted for publication in Nature Com.

We hope we have sufficiently address these critiques.

Reviewer #2 (Remarks to the Author):

In this manuscript Ortlund and colleagues describe the identification of novel binding sites for the DNA binding domain (DBD) of the glucocorticoid receptor. This is the latest contribution to a larger body of work from this lab attempting to map the GR DBD binding sites and their associated functions seeking to define a novel paradigm for GR action which moves past the time honored but imperfect activation vs repression paradigm for GR. In the opinion of this reviewer the work represents another chapter in understanding the role of NFKB binding sites in GR signaling. Yet the impact of the conclusions of this paper is not substantial enough to warrant publication in Nature Communications. The authors could consider some suggestions below to add impact, or if they wish, the contents of this manuscript should definitely be published in another journal.

The science is excellent. The authors show a deep understanding of GR and its associated signaling / binding sites. The identification of the kBREs is notable, and the crystallography experiments are well done. The NMR work clearly supports the X ray data regarding the kBRE binding. Yet it seems the previous publication in Nature Structural and Molecular Biology in 2013 where the nGRE sites were similarly identified led to a very similar set of experiments examining a subset of the kBREs discussed here. Many of the figures and tables follow the example of this earlier publication. The authors do demonstrate that the kBREs investigated are not 'tethered' to the DNA sequence rather a direct interaction has been noted for these response elements. The direct binding of the GR mutants studied is observed for SOME kBRE containing promoters but the authors have carefully selected them to avoid effects by stimuli (TNF) or glucocorticoids (dexamethasone, triamcinolone acetonide) despite the statement that a large number of these genes are affected by both agents with many under kBRE control. This is perhaps of greatest concern: Identification of these sites selectively and defining the associated crystal structure is impressive yet any association of these binding sites with the stimuli or steroid would be highly impactful. Why for example would one be interested in GR binding to any sequence of DNA in the absence of ligand? Is there a novel constitutive component of GR activity that is not characterized in the isoforms of this receptor with a missing LBD?

This is a very interesting question that we are pursuing separately from this manuscript. We tried making stable cell lines that knockdown GR with shRNAs and there was no knockdown of the protein even though the mRNA was lower. To answer this question, we would probably need to do Crispr-CAS9 mediated knockout of GR and then RNAseq. Given Gordon Hager's estimates that the full GR response involves only 10% occupancy of GREs, we suspect very little nuclear GR is required for some activity.

Steps towards resolution of ligand, stimulus, and function at the level of transcription are prized goals for nuclear hormone research. The authors have mentioned so called differentiated ligands in their body of work that have been examined for decades. Would it be worthwhile to characterize one of these (compound A?) as a start towards understanding the gene response of non steroidal ligands showing differentiated function? Efforts to this end would add impact and be highly attractive to the readers of Nature Communications.

We agree that this would be highly impactful and in response to this question, we performed a series of experiments examining the mechanism of CpdA action (below). Briefly, we found that Compound A only inhibits inflammatory gene expression at 10 µM, and this effect is not reversible by RU486.

MCF-7 cells show repression of *IL6*, *IL8,* and *ICAM1* in response to 100 nM Dex (**Fig. 7a**). In MCF-7 cells stimulated for 4 h (see below), 1 µM compound A was pro-inflammatory on IL8 and perhaps IL6, but 10 µM compound A had strong anti-inflammatory effects. Compound A has a slightly higher GR-binding affinity than Dex (*De Bosscher K et al. PNAS 2005*). Therefore, it is remarkable that compound A did not suppress TNF-induced cytokine expression at lower doses.

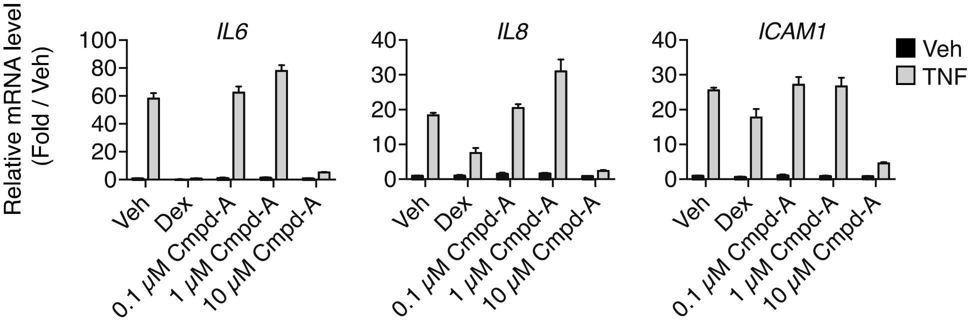

In cells pre-treated for 1 h with 5 µM RU486, which blocked both the GR agonist activity and anti-inflammatory effect of 100 nM Dex, 10 µm compound A was still able to suppress TNF-induced cytokine expression, and induce *GILZ* expression (see below).

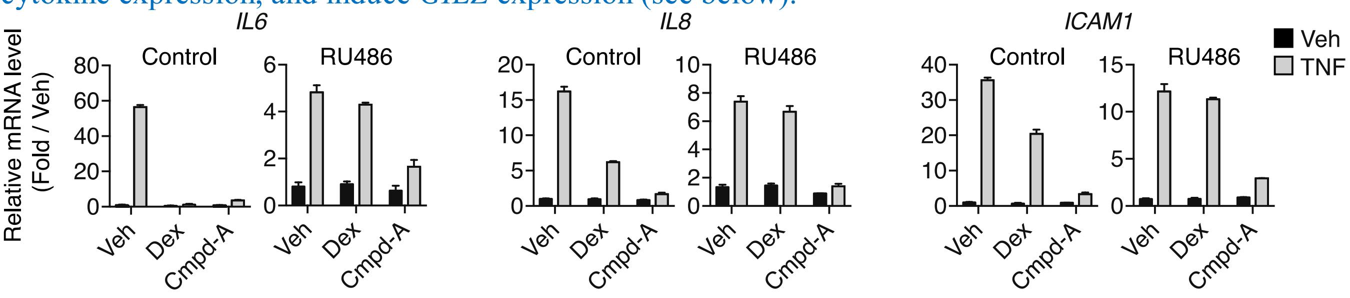

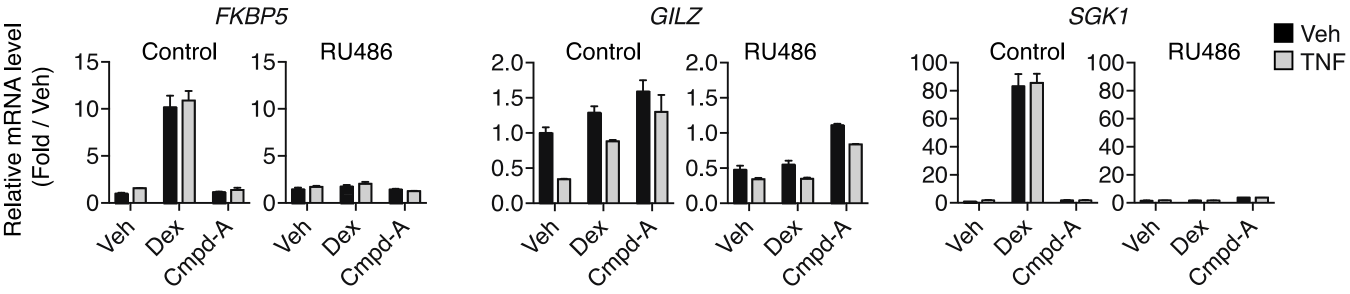

Further increasing the concentration of RU486 up to 100 µM was not sufficient to block the anti-inflammatory effect of 10 µM compound A (see below).

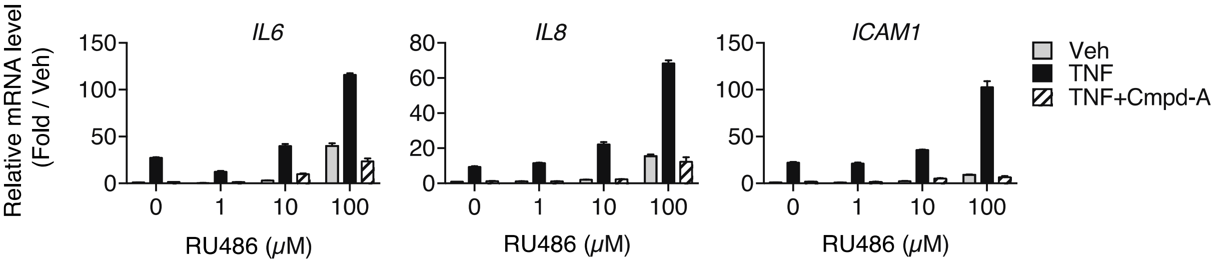

These results suggest that compound A inhibits inflammation through a protein other than GR. Therefore, we will examine the mechanisms underlying compound A activity in greater detail in a separate upcoming manuscript.

All of that said the body of work from these authors has been impressive. Their efforts have provided a novel paradigm for GR monomeric vs dimeric gene regulation that could lead to a novel paradigm for GR modulation by NCEs. This manuscript offers another short chapter in the characterization of one such response element associated with some NFKB responsive genes and as such adds to the impressive story of this investigation. However the impact of this particular chapter is eclipsed by earlier

publications where the process, results and expectations were similarly described for another DNA response element. Hence my recommendation to enhance this work or publish elsewhere.
I have enclosed a proof written version of the original submission for the benefit of the authors. Perusal of this annotated pdf should assist the needed revisions and point out simple corrections. The authors are encouraged to check their reference management software as there are many duplicated citations in the reference section (12 & 65, 19&22, 29 & 30).

Thank you for your detailed recommendations. In response to your suggestions, we have now added an additional figure 7 showing the results of a small-scale shRNA screen, which showed that a number of different coregulators are required for GR-mediated suppression of IL6. We performed ChIP assays for two of the coregulators, which showed that they are recruited to IL6, but differently to IL8 and ICAM, suggesting differences in timing or which coregulators are required. Lastly, we performed ChIP assays with WT or DNA binding dead mutant GRs in 293T cells and showed that SRC2 recruitment to the IL6 gene required GR DNA binding. We suggest these additional experiments provide sufficient novelty, as we believe this is the first demonstration that there are multiple different coregulators required for GR-mediated inhibition of inflammation for specific loci.

Reviewer #3 (Remarks to the Author):

The team of Eric Ortlund reports on the presence of cryptic GR binding sites positioned at genomic NF-kappaB binding sites, and proposes this mechanism as a likely contributor to the repression of inflammatory genes by glucocorticoids. In line with earlier findings of Uhlenhaut et al., who reported on tethering being unlikely as a mechanism to explain GR-induced gene repression mechanisms at inflammatory gene promoters, the current study has aimed to moved further and to propose how this alternative mechanism may work. Regardless of how attractive and plausible this novel hypothesis may be, the molecular biology part of the study unfortunately has several shortcomings, with data presented in an unclear manner and/or missing a fair amount of control experiments to scientifically back up the statements, and to unambiguously draw solid conclusions. In many experiments, statistical analyses are lacking.
Major comments
1) An important comment is why authors have used throughout their study, for many experiments only GR-DBD while they clearly show that they also have a baculovirus-expressed full length GR at over 99% purity. To strengthen their statements and hypothesis, authors should be able to recapitulate their findings with this full length GR, instead of with a partial GR moiety, which may not reflect a physiologically relevant situation.

Unfortunately, the full-length protein is very difficult to express and purify. We reached out to David Bain, who produced this protein for us, and he confirmed that producing sufficient quantities of GR for these studies is not possible. Further, ligand is required during expression and purification to stabilize the protein, making experiments with the unliganded receptor technically unfeasible. We also spent the last year trying to make apo full-length GR in our lab using mammalian cells. Again, we are unable to generate sufficient quantities for our biochemical studies. However, we do show that the isolated DNA Binding Domain approximates the behavior of full-length protein and all the in-cell data were generated with full-length receptor.

2) A second important comment is that authors should back up their findings by including a gelshift analysis, using both unliganded versus liganded recombinant FL GR, as well as a control versus dex-

activated cell lysate, on these isolated cryptic GR binding NFkappaB elements. Supershift analysis using an antibody against GR should demonstrate GR presence.

We agree with the reviewer's comments (1 and 2) that full-length protein is the most desirable construct for probing the function of GR. Unfortunately, this protein is very difficult to express and purify, and only limited amounts are available for *in vitro* experiments. Accordingly, we have used full-length to show that full length GR does bind κBREs in a similar manner as the isolated DBD (New Figure 2d), and have used the isolated DBD in other *in vitro* assays. However, all in-cell assays (ChIP, reporter assays) use the full-length protein, which we believe is demonstrates the applicability our findings to in-cell biology.

3) Could GR bind to these cryptic sites in absence of NFkappaB? In other words, can a mutant site be identified that eradicates NFkappaB binding, yet retains GR binding?

As we are in interested in the GR binding footprint here, we address the inverse of this question in Figure 6d: we have identified mutations in the *IL8* κBRE that retain activation by TNF-α yet do not permit repression by GR.

4) Around the world, in different labs, many commercially available NF-kappaB promoters linked to luciferase are being used. Have authors checked whether these also contain the cryptic GR binding sites?

This is an excellent question which we had not considered. The pGL4.32[luc2P/NF-κB-RE/Hygro] vector (available from Promega) contains three tandem repeats of the κBRE sequence GGG**AATTT**CC, which does contain the cryptic GR binding site (bolded). The Affymetrix NF-κB reporter includes five repeats of the same sequence.

5) P3 and Fig S1a. The authors claim that GR Ser425Gly, a mutant that fails to bind DNA and to interact with NFkappaB, is unable to repress the constitutively active IL-8 promoter.
a) I fail to see this: in Fig S1a, as compared to control levels, there is indeed a larger difference as compared to GR wildtype, yet a mild, yet consistent, inhibitory response is visible also for GR S425G? Please clarify.

We have clarified this in the main text; the reviewer is correct. A mild inhibitory effect is visible, and we have updated the text to reflect that the mutation hinders (but does not ablate) the ability of full-length GR to repress the *IL8* reporter. It is important to note that the Ser425Gly mutant is not DNA-binding dead, but is less effective at DNA-dependent transrepression; thus, this defect can be bypassed by increasing the amount of S425G GR in the cell (Hudson et al, *PNAS*, 2016).

b) Transfection procedures may also cause a minimal activation of NFkappaB, and the mutant may rather be compromising NFkappaB binding instead of killing the interaction completely, which might explain residual inhibitory effects. Authors should check whether mutating the NFkappaB binding site, would still yield suppression or not with both constructs.

Both constructs are transfected, and should therefore have the same effect on NF-κB activation. HeLa cells are used for these experiments, and endogenous GR may explain the mild repression; the S425G mutant is not compromised in its interaction with NF-κB (Figure 1d). In addition, we also mutate the NF-κB binding site in Figure 6d; mutation of the spacer between the NF-κB footprint ablates the ability of GR to repress TNF-α-induced activation.

c) Statistical analysis is lacking for control versus transfected levels with S425, or wt, which makes the conclusion anyway uncertain. Number of independent experiments is missing.

We have added the number of experiments to Figure S1. Additionally, we now show expression of WT GR and the S425G mutant in Figure 1a; moreover, transfection of an identical amount of WT or S425G GR plasmid also drives the same amount of activation from a 3X (+)GRE reporter plasmid:

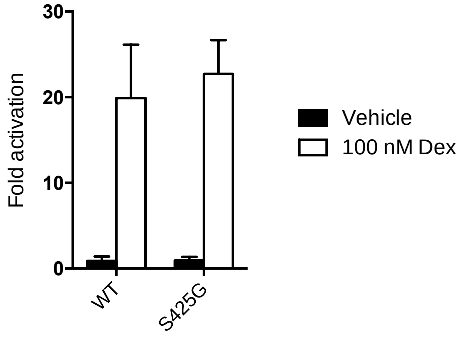

d) If statistics would reveal there is indeed a residual inhibitory effect, then what could be the explanation (in case b) could be ruled out)? Can the presence of other TF binding sites that may still be present in this promoter, explaining this remaining ability to repress?

Please see our responses above. S425G does have a mild inhibitory activity. In addition, if there is some inhibitory effect of S425G interacting with NF-kB (as the reviewer suggests in part b), we show in Fig. 1d that S425G interacts with p65, just like WT.

e) This experiment further lacks an essential control: a Western analysis of transfected protein levels for both GR wt and GR S425G and a loading control should accompany this result.

We have now included a Western analysis of WT and S425G protein levels in Figure 1.

f) Further, the authors have made the 400bp IL-8 reporter "constitutively" active by flanking a constitutively active viral minimal promoter, but it was not shown whether or not this construct remains functional and still behaves as expected when induced with an inflammatory signal, IL-1beta or TNF.

The goal of this experiment was to investigate the ability of GR repress at this promoter without the presence/ induction of NF-kB by means of a straight-forward biochemical assay. The constitutively active plasmid is a tool that enables us to test question without (or at least by minimizing) confounding factors. We included 400bp of the IL-8 reporter to ensure that we would see a meaningful response as opposed to including only short elements containing the kBRE or putative GRSs. Shlyueva et al (PMID 24685159) showed – for the Drosophila ecdysone receptor – that ectopic reporter assays recapitulate cell type-specific ecdysone-induced enhancer activities that correlated with transcriptional changes of the neighboring genes, i.e. likely reflecting physiological enhancer activities when extended enhancer sequences are included (i.e. >500bp or so).

g) It is not stated in the methods whether or not for this experiment, the total amount of transfected DNA has been kept constant (only for FigS2).

For the transfection experiments in Figure S2, all amounts of reporter DNA transfected are kept constant. We have clarified this in the methods.

6) Fig. S1b and c: statistical analysis is missing. N=?

This has been added to the legend.

7) Fig S1b and c: authors should explain the implication of these results in more detail in the results section, for the separate graphs. S1c is difficult to interpret, it would be easier to interpret and allowing direct comparison, when results for GRwt are also depicted in FigS1b.

This is a good suggestion; we have added a sentence to the results section (p. 3) concerning the implication of these results and added additional text to the Figure S1 legend. The inability of the GTG3A mutant to bind 'traditional' GR response elements was originally used as evidence for DNA-independent repression of NF-κB. However, the relative affinity of the GTG3A mutant for alternate DNA sequences - such as the nGRE - led us to seek a DNA-dependent mechanism of GR to inhibit NF-κB.

8) Fig S1c: the Y-axis states "% compared to wt", but the numbers on the graph do not seem to indicate this.

As stated above, we have updated the legend to Figure S1 to clarify this figure.

9) P3. The results section mentions data on 2 GR mutants, A458T and GTG3A, yet the referencing is unclear and the graph only shows results with GTG3A. Are data in the graph missing?

We apologize for the confusion. The GRdim mutation (A458T) has been shown by other groups to be able to repress NF-κB. As A458T was originally thought to be unable to bind DNA, this mutant was used as evidence for DNA-independent repression of NF-κB by GR. However, recent work by Presman et al. and Lim et al. (see references) show that the GRdim mutant is capable of binding DNA.

10) P3. The final conclusion from FigureS2 is not novel and already supported in 1997 by the work of Lidén et al, as well as of Wissink et al.

The experiments by Liden et al. (*JBC* 1997) mutated rat Arg488 and Lys 490 which do not directly contact DNA. To make a mutant GR that cannot bind DNA, we instead mutated the Arginine and Lysine within GR's DNA binding helix (residues 460 and 465 in the rat GR).

11) Figure 1 (c-e). Authors rely on results of another study. How efficient was the knockdown of p65 (this should at least be stated)? Lack of signal for p65, at sites where a signal for GR is present, could have multiple reasons, e.g. the remainder p65 epitope could be masked (as could be expected when a tethering takes place), or either for the depicted sites the NFkappaB binding site could be occupied by other NFkappaB family members. The discussion on this should therefore be better nuanced.

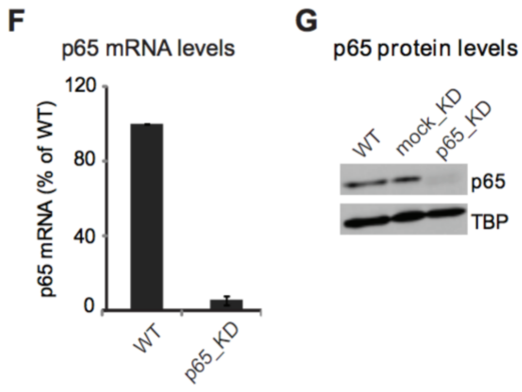

We have placed here the p65 knockdown results from Rao et al (Supplementary Figure 2). We have updated the legend of Figure 2 to discuss that knockdown was efficient. While it is possible that GR could mask the p65 epitope, Rao et al. also show multiple sites with GR and p65 co-occupancy. However, the reviewer is correct that another NF-κB subunit could be present; we have updated the text on page 4 to clarify that p65 is one subunit of NF-κB.

12) Figure 1f. Please indicate in what region the ChIP primers were chosen, and where the respective TF binding sites are localized.

In the *IL6* promoter, the NF-κB response element is 15bp upstream (-51) of the transcription start site (transcript *IL6*-201). The ChIP primers in the indicated figure span -52 to +51 of the *IL6* promoter.

13) Figure 1f. Is this performed in HEK293T (which contain very little GR basally, and are therefore often used as a cell line to check GR mutant functionality) or on GR-transfected HEK293T? This is unclear.
Authors state further that there are no potential tethering factors present, yet a signal for Fra 1-2 is shown, so please clarify why this is disregarded?

Thank you for pointing this out. We have indicated in the figure legends the type of cells that were used. We agree that the AP-1 subunits, Fra-1 and Fra-2, can form heterodimers with Jun family proteins, and we realize that the promoter region of cytokine genes such as *IL6* contains binding sites for several other TFs, many of which GR could potentially be tethered to. However, determining the repertoire of TFs recruited to *IL6* promoter distracts from the main point of the manuscript. Therefore, we have revised the manuscript to focus on the GR ligand/Dex-dependent effects on NF-kB activity, a cornerstone for clinical use glucocorticoids as anti-inflammatory agents.

We added **Figure 7** (new), which clarifies the effects of Dex on NF-kB target genes without any overexpression. Dex suppressed expression of NF-kB target genes to different extents in MCF7 cells. Without TNF stimulation, Dex increased recruitment of endogenous GR to these genes, as shown below (**Fig. 7b**). TNF stimulation induced recruitment of p65 and reduced Dex-dependent recruitment of GR, consistent with competition between GR and NF-kB for nGRE/kBRE binding. However, Dex also blocked TNF-dependent recruitment of p65 at *IL6*, but not at *IL8* or *ICAM1*, indicating that additional factors are required to explain this anti-inflammatory action of Dex.

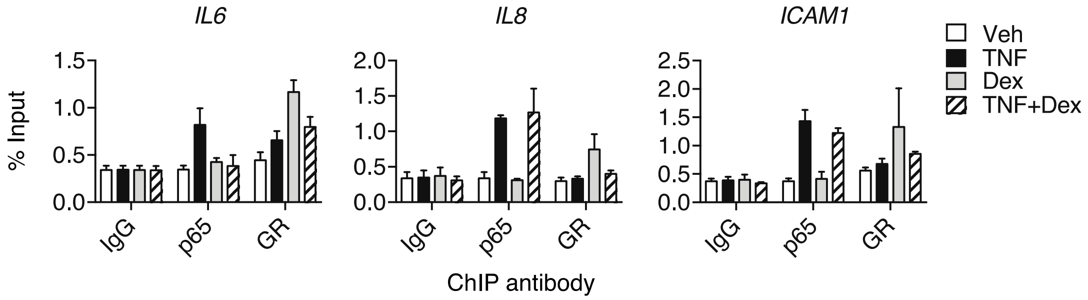

We suspected that like the estrogen receptor (Nwachukwu JC et al. *elife* 2014), GR also requires transcriptional coregulators to mediate ligand-dependent repression of NF-kB. Using the same siRNA screen previously described (Nwachukwu JC et al. *elife* 2014), we identified a subset of coregulators required for Dex-dependent repression of *IL6* in MCF7 cells (**Fig. 7c**) and show that some of these coregulators are recruited to nGREs/kBREs in Dex/GR-dependent manner (**Fig. 7d–f**).

14) Figure 1f. Authors must also realize that in the IL-6 promoter other TF binding sites are present which could contribute, eg. CREB (which is also a potential GR-tethering factor).

We agree, please see comment 13 above.

15) Figure 1g. the most important control is missing and this is p65.

Thank you for pointing this out, we now show recruitment of p65 in the new **Figure 7b**.

16) Figure 2d. Corresponding controls for this experiment are missing, i.e. the loading control for p65 overexpression and also the negative antibody control for the immunoprecipitation (IgG control). Plus, the loading control for HA-GR is also missing and it cannot be extrapolated from Figure 2a, since this is another experiment (inductions at different times and in absence of p65 overexpression).

We added the loading controls shown below for p65 and HA-GR co-expression in 293T cells (**Fig. S5b**)

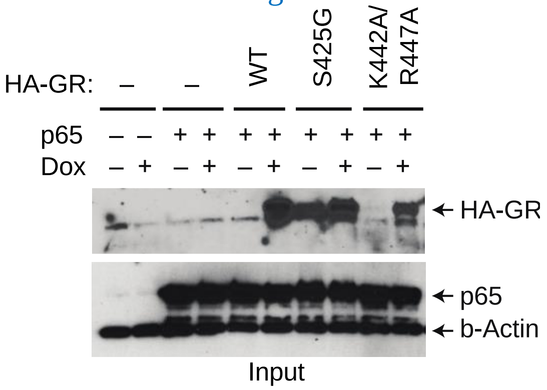

We also added the negative antibody control co-IP experiment using normal rabbit IgG (shown below) to **Figure 2d**.

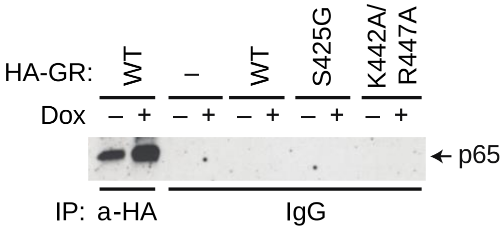

17) Figure 2b. What is the % recruitment over input?

The Y-axis of ChIP data are now shown as % input throughout the manuscript.

18) Figure 2b. A negative control of recruitment levels in absence of overexpressed GR is missing.

We showed that in this system, HA-GR overexpression is induced by Dox (**Fig. 2a**), and the recruitment of levels detected by ChIP using anti-HA antibody, in the absence versus presence of Dox are shown in the first two columns of **Figure 2b-c**.

Also, why is there a strong signal of HA-GR recruitment for GILZ, SGK1 and FKBP5 in absence of ligand? Is this the consequence of the overexpression?

The recruitment profiles of native GR in MCF7 cells (**Fig. 7**) and overexpressed HA-GR in 293T cells (**Fig. 2b-c**) are different, suggesting that overexpression alters the GR recruitment profile. Also, we detected endogenous GR at *FKBP5* in steroid-deprived MCF-7 cells stimulated with 100 nM Dex, but not in vehicle-stimulated cells (see below). Therefore, it is likely that the recruitment profile of HA-GR in 293T cells is a consequence of overexpression.

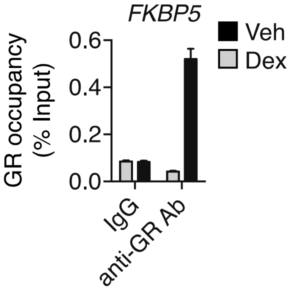

Connected herewith, in Figure S5, how do basal mRNA compare, i.e. are these genes also basally activated in absence of DEX?

We have revised the manuscript to focus on GR ligand-dependent effects on NF-kB activity. The basal mRNA levels of classical GR-target genes in 293T cells shown in **Figure S5a** (steroid-deprived without Dex stimulation) are shown below.

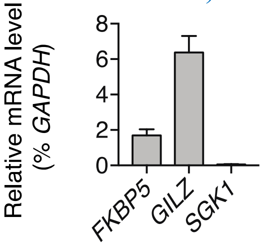

We also compared mRNA levels in 293T cells transfected as described in **Figure 1a** with empty vector, HA-GR-WT, or HA-GR-S425G. As shown below, overexpression of HA-GR selectively increased the basal mRNA level of *IL6* and *ICAM1*, and this effect was reduced to different extents by the S425G mutation (n=8, *$p_{adj} < 0.05$, 2-way ANOVA, Tukey's multiple comparisons test). Without ligand stimulation, GR overexpression did not affect basal mRNA levels of the classical GR target genes, *FKBP5*, *GILZ*, and *SGK1*, in steroid-deprived 293T cells.

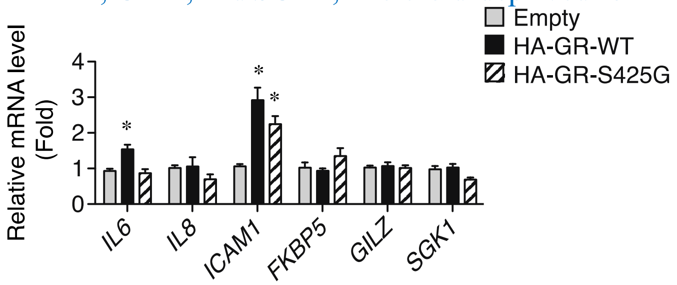

Plus, in Figure S5, is HA-GR here actually overexpressed or not? (HEK293T, low in endogenous GR, are in other experiments always supplemented with GR).

We have clarified in the figure legend (**Fig. S5a**) that the 293T cells were not transfected (i.e. GR was not overexpressed).

19) Figure 2b. Why is the signal consistently lower for recruitment of HA-GR at IL-6, ICAM and IL-8 promoters, upon activation of GR with DEX (third lanes vs. second lanes). This would almost indicate that a ligand-independent binding event is being favored at these sites? Please clarify.

It is likely that the recruitment profile of HA-GR in 293T cells is a consequence of overexpression (please see comment 18, above). Dex induces recruitment of endogenous GR to these sites in MCF7 cells (**Fig. 7b**).

20) Figure 2b. Recruitment of HA-GR at the IL-6 promoter with GR S425G. Why is the third lane of the hatched bars missing and hence, why is this signal not consistent with the other promoters?

Thank you for pointing this out. We have corrected this mistake.

21) Figure 2b, pertaining to the four questions above, statistics are missing (n=3 so it should be possible to do this analysis at an averaged figure result) so it is difficult if not impossible to draw definite conclusions from these results.

We have revised (new) **Figure 1b-c**, to clarify that the comparisons between WT and mutant HA-GR. Most of the differences between the WT and mutant HA-GR recruitment were significant; therefore, we have indicated the non-significant differences (2-way ANOVA, Tukey's multiple comparisons test).

22) Figure 2b, assuming changes are statistically significant, how do authors explain that the highest recruitment of HA-GR at IL-6, ICAM and IL8 promoters in presence of DEX/TNFalpha. Can a TNF-induced modification lie at the basis, or can a classic tethering mechanism

This observation is consistent with increased chromatin accessibility from the TNF-induced recruitment of p65 and assisted loading of HA-GR directly to DNA in TNF-treated cells. However, the recruitment profile of HA-GR is probably a consequence of overexpression (please see comment 18, above), as TNF

stimulation generally reduced Dex-dependent recruitment of endogenous GR to the cytokine genes in MCF7 cells (**Fig. 7b**).

23) Figure 6d. Y-axis is unclear, what is measured? Luciferase? Not sure conclusions are valid here, as data can also oppositely be interpreted that the TNF-inducing ability is lower for the mutated sites, without affecting the gene repressive capacity of dex-activated GR.

This is indeed a basic luciferase reporter, with 400 bp of the WT *IL8* promoter (or indicated mutations) upstream of the luciferase gene. We have updated the figure legend to indicate that luciferase activity is being measured. We agree that the double mutation does affect the ability of TNF-α to induce the element, although this is not unexpected given that fact that we are mutating the NF-κB response element. However, repression - as measured by the reduction in luciferase activity from TNF-α levels to Dex + TNF-α levels - is decreased by the mutations.

24) Figure S4d: have authors checked the binding behavior of the reverse control experiment, i.e. p65 DBD in this assay?

In response to this suggestion, we have recombinantly expressed p65 and used fluorescence polarization to test its ability to bind NF-κB response elements. For example, the *TSLP* κBRE data is shown below. However, we are not sure that this adds to the manuscript as multiple crystal structures and binding assays of NF-κB to its response elements are reported in the literature (Zabel et al., *JBC* 1991, Chen et al., *Nature* 1998, etc.).

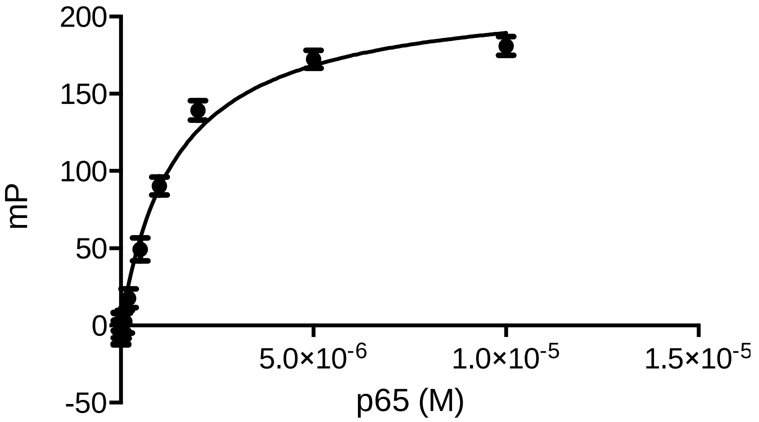

25) In the discussion section, p8 second paragraph, authors list several mechanisms that do not have to be exclusive but may co-exist with the novel reported mechanism. They state that ChIPseq studies have not yet validated a widespread occupancy of IR-GBSs by GR, potentially due to a lower affinity for GR compared to dimeric GBS elements. Surely, the same argument would also be the case for protein-protein interactions, as in the case of the classic tethering model, and a failure to find support for this model by a classic ChIP approach ChIP could, by extension, also be caused by variation in experimental ChIPping conditions, as it is more difficult to trap these interaction types as opposed to a direct protein-DNA interaction. This argument should also be integrated in the discussion.

We have revised and streamlined the discussion to highlight direct-DNA may be occurring on a subset of kBREs, and that tethering via direct and indirect interaction with p65 complexes is also likely occurring in GC-mediated transrepression which is technically difficult to assess.

Minor comments

1) Overall, Figures are not sufficiently self-explanatory and legends are therefore rather extensive.

2) In the abstract authors state the cryptic sites are recognized between the binding sites of NF-kappaB, but this may confusingly also be interpreted as “besides” multiple NFkappaB binding sites in a given promoter, and not at the actual NFkappaB binding site. Please revise.

This is an excellent observation. We have updated the wording in the abstract to make clear GR binds *within* the NF-κB response element.

3) Although the PDB code is given in the legend, in Fig 1B. it could be just defined from which gene promoter the canonical GBS originates.

The paper reporting this structure (Meisjing et al., *Science* 2009) refers to this site as the ‘Cgt’ GBS. However, as Cgt is not an official gene symbol, we prefer to simply cite the PDB code here.

4) Ref22 should be deleted as it is the same as ref 19.

We apologize for the reference errors; these were inadvertently duplicated during editing by multiple authors.

5) Fig S1a. How are graph results calculated: if % of control response, should the first bars not be set at 100?

We thank the reviewer for noticing this! We ran parallel experiments on this plate and normalized to the wrong control (a nGRE control instead of the appropriate IL8 SV40 vector control). We have normalized to the appropriate IL8 control and have updated the figure. The bars in Fig. S1a are now at 100% of control and the statistics/ conclusions were unaffected.

6) Figure 1 (c-e), in the legend ref 25 is wrongly cited. Please check.

We edited the legend removed this reference since the data sets used for this analysis are detailed in the methods.

7) Figure 1 (c-e). For the read in presence of activated p65, i.e. the controls, where do the p65 peaks localize? Including these results would be helpful.

We agree and have added the p65 ChIP track with TNFα treatment.

8) Figure 1f. “in some instances”. Please rephrase, for the sake of clarity.

We have removed this confusing phrase.

9) Figures 1f and g are both ChIP results, why are different Y-axis used? ChIP binding results should be depicted as %binding over input control.

Per the Reviewer’s suggestion below (and other reviewer comments regarding a lack of need for these data), we have replaced these panels with the GR and MR DBD binding data.

10) Figure S4 seems as a key result. Why do authors present this as Supplementary data?

We have moved these data into Fig. 2 as panels e and f per the Reviewer's request.

11) P 8. First paragraph, last sentence. Part of the sentence is missing to be understandable. Please fix.

We have corrected this sentence.

12) Figure 5e. Define green in legend.

We have corrected the legend.

13) Figure 6c. What assay has been done here? Please specify.

We measured GR DBD binding to DNA using fluorescent polarization. The data are shown in bar graph form to permit a quick characterization of affinity. Affinity is derived from fitting the binding curve to two-site equation, which generates a high- and low-affinity binding affinity. This mirrors what is seen for nGRE binding (Hudson et al, PNAS, 2016; Hudson et al, NSMB 2013).

14) Ref 29 and 30 are duplicates.

We have removed the duplicate reference.

REVIEWERS' COMMENTS:

Reviewer #2 (Remarks to the Author):

This revision should be a welcome addition to Nature Communications. All of the concerns expressed in my original comments have been addressed, and many many additional experiments now address additional concerns from other referees. I was very pleased to see that the experimental details requested and additional rigor has now been applied resulting in a significantly improved manuscript. I recommend publication after perusal by copy editors who will address lingering manuscript errors (Figure caption 2: the MR curves should be labeled panel f - not c as in the pdf).

When I first reviewed this paper i felt the science was impressive but there was a lack of attention to detail and the manuscript had contributions from many sources. I suggest future efforts welcome the contributions for multiple authors but the corresponding author should also act as editor prior to final submission.

I recommend publication

Reviewer #3 (Remarks to the Author):

Authors have adequately and satisfyingly addressed most of my queries. Only two issues remain that, if possible, I would like to see addressed/further clarified. My question 3 was answered a bit beside the point. I asked whether unambiguously could be established GR may bind to these cryptic sites in absence of NFkappaB binding? In other words, can a mutant site be identified that eradicates NFkappaB binding, yet retains GR binding? Authors answered: “As we are in interested in the GR binding footprint here, we address the inverse of this question in Figure 6d: we have identified mutations in the IL8 kBRE that retain activation by TNF-ɑ yet do not permit repression by GR”. However, my question pertained binding events not activation/activity events and I still believe this is a relevant question, that can be addressed by using EMSA and NFkappaB and GR from cellular lysates.
The second issue is on my first major query and that was why authors have used throughout their study, for many experiments only GR-DBD while they clearly show that they also have a baculovirus-expressed full length GR at over 99% purity. I am satisfied by the answer however, and I sympathize with the authors having spent a long time in trying to get the material. However, I strongly feel authors should include this in the body of the manuscript. Other researchers would now wrongly get the impression that the assays I asked for in my query 1 and 2 are perfectly feasible, whilst they are not.
Thirdly, this is merely a comment. Authors state on the experiment for Rev2 that CpdA does not bind GR based on the RU486 experiment. This is perhaps not the only explanation possible, as RU486 has a binding mechanism that may be different from nonsteroidal compounds. Competition is only to be expected for similar binding modes.

Reviewer Response

Reviewer #2 (Remarks to the Author):

This revision should be a welcome addition to Nature Communications. All of the concerns expressed in my original comments have been addressed, and many many additional experiments now address additional concerns from other referees. I was very pleased to see that the experimental details requested and additional rigor has now been applied resulting in a significantly improved manuscript. I recommend publication after perusal by copy editors who will address lingering manuscript errors (Figure caption 2: the MR curves should be labeled panel f - not c as in the pdf).

We have corrected this and all other typographic errors.

When I first reviewed this paper i felt the science was impressive but there was a lack of attention to detail and the manuscript had contributions from many sources. I suggest future efforts welcome the contributions for multiple authors but the corresponding author should also act as editor prior to final submission.

I recommend publication

Reviewer #3 (Remarks to the Author):

Authors have adequately and satisfyingly addressed most of my queries. Only two issues remain that, if possible, I would like to see addressed/further clarified. My question 3 was answered a bit beside the point. I asked whether unambiguously could be established GR may bind to these cryptic sites in absence of NFkappaB binding? In other words, can a mutant site be identified that eradicates NFkappaB binding, yet retains GR binding? Authors answered: “As we are in interested in the GR binding footprint here, we address the inverse of this question in Figure 6d: we have identified mutations in the IL8 kBRE that retain activation by TNF-α yet do not permit repression by GR”. However, my question pertained binding events not activation/activity events and I still believe this is a relevant question, that can be addressed by using EMSA and NFkappaB and GR from cellular lysates.

Our apologies! We now understand this inquiry – can GR bind to these cryptic sites in absence of NFkappaB binding? EMSA experiments would indeed provide additional evidence to support direct binding in the absence of NFkappaB; however, we believe that we have evidence for binding in the absence of NFkappaB in the following experiments:

1) Fluorescence polarization in vitro including only purified GR and these DNA elements
2) Crystal structure of GR on these elements in the absence of NFkappaB
3) NMR Foot printing data for both the DNA and the GR DBD using purified components

4) ChIPSeq experiments in cells upon p65 knockdown showing GR occupies these sites in the absence of NFkappaB

We are also unsure how we would exclude NFkappaB as a factor if we used cellular lysates.

The second issue is on my first major query and that was why authors have used throughout their study, for many experiments only GR-DBD while they clearly show that they also have a baculovirus-expressed full length GR at over 99% purity. I am satisfied by the answer however, and I sympathize with the authors having spent a long time in trying to get the material. However, I strongly feel authors should include this in the body of the manuscript. Other researchers would now wrongly get the impression that the assays I asked for in my query 1 and 2 are perfectly feasible, whilst they are not.

Thank the reviewer for these comments agree that we need to better explain why we performed only a limited number of in vitro binding experiments with full-length GR. We have now clarified this in the manuscript (below).

"Remarkably, the affinity of full-length GR for the two elements was very similar, with K_ds of 34 and 51 nM for the *SGK1* (+)GRE and the *IL8* κBRE, respectively. We were unable to purify enough full-length GR to test additional DNA elements so we also tested the ability of the isolated GR DBD to bind the *IL8*, *CCL2*, and *PLAU* NF-κB response elements *in vitro*."

Thirdly, this is merely a comment. Authors state on the experiment for Rev2 that CpdA does not bind GR based on the RU486 experiment. This is perhaps not the only explanation possible, as RU486 has a binding mechanism that may be different from nonsteroidal compounds. Competition is only to be expected for similar binding modes.

Great point. This is certainly a possibility if CpdA hits the AF-2 or a surface other than the canonical ligand binding pocket.
